# Direct synthesis of a semiconductive double-helical phosphorus allotrope in a metal-organic framework

**DOI:** 10.1038/s41467-025-55999-4

**Published:** 2025-02-12

**Authors:** Sergei A. Sapchenko, Rodion V. Belosludov, Inigo J. Vitoria-Irezabal, Ivan da Silva, Xi Chen, George F. S. Whitehead, John Maddock, Louise S. Natrajan, Meredydd Kippax-Jones, Dukula De Alwis Jayasinghe, Carlo Bawn, Daniil M. Polyukhov, Yinlin Chen, Vladimir P. Fedin, Sihai Yang, Martin Schröder

**Affiliations:** 1 Department of Chemistry, University of Manchester, Manchester, M13 9PL UK; 2 Institute for Materials Research, Tohoku University, Sendai, 980-8577 Japan; 3 ISIS Facility, STFC Rutherford Appleton Laboratory, Oxfordshire, Oxfordshire, OX11 0QX UK; 4 College of Chemistry and Chemical Engineering, China University of Petroleum (East China), Qingdao, 266580 PR China; 5 Nikolaev Institute of Inorganic Chemistry SB RAS, 3 Lavrentiev Ave, Novosibirsk, 630090 Russian Federation; 6 Faculty of Natural Sciences, Novosibirsk State University, 1 Pirogov Str., Novosibirsk, 630090 Russian Federation; 7 College of Chemistry and Molecular Engineering, Beijing National Laboratory for Molecular Sciences, Peking University, Beijing, 100871 PR China

**Keywords:** Metal-organic frameworks, Organic-inorganic nanostructures, Photocatalysis

## Abstract

There remains much ambiguity regarding the structure of red phosphorus. We report the adsorption and photo-polymerisation of P_4_ molecules encapsulated in an indium(III)-based metal-organic framework to afford a double-helical chain composite comprising of [P_8_] units. The similarity between the Raman spectrum of bulk red phosphorus and of the metal-organic framework – (P_8_)_n_ adduct suggests the presence of such helical chains in the structure of amorphous red phosphorus. This provides crystallographic evidence of the structural building blocks of the red phosphorus allotrope stabilized within the pores of a metal-organic host. The (P_8_)_n_ inclusion compound is an air-stable semiconductor with a band gap of 2.3 eV, which is relevant for gas detection and photo-catalysis. We demonstrate that this phosphorus adduct demonstrates a 10-fold increase in conversion in the oxidation of methyl orange dye compared with the parent metal-organic framework material.

## Abstract (web summary)

The structure of red phosphorous remains poorly elucidated. Here, the authors report the adsorption and photopolymerization of P_4_ encapsulated in a metal-organic framework to obtain a double-helical chain composite comprising of [P_8_] units in the porous material.

## Introduction

Phosphorus is remarkable for the structural diversity of its allotropes, but only a few of them are chemically stable and possess desired properties. The molecular forms of low nuclearity species are unstable, with white phosphorus P_4_ self-igniting in air, and P_2_ and P_6_ species existing only in the gas phase at high temperature^1,2^. In contrast, polymeric red, violet and black phosphorus can be handled readily under ambient conditions. Phosphorene, a monolayer of black phosphorus, was recently demonstrated to be a semiconductor with a tuneable bandgap^3,4^. However, these sheets of black phosphorus are not stable towards a combination of moisture, oxygen and light. Another drawback is the high energy consumption during the synthesis of this allotrope from P_4_ at 1.3 GPa and 200 °C^5^. Another important and stable allotrope is red phosphorus, which is more stable than black phosphorus with a wide bandgap of up to 2.4 eV^6^. Decreasing the bandgap of red phosphorus-based materials is a challenging task, and extensive theoretical and synthetic investigations have been conducted to develop new polymeric forms of phosphorus with improved stability and physiochemical properties.

Von Schnering and Baudler have demonstrated^7,8^ various types of clusters of phosphorus, including the structural elements of black phosphorus. *Cis*- and *trans*-linear chains, helical and double-helical chains containing [P_8_] cages directly bound to each other have been predicted recently to be more stable than P_4_^9^. A phase-pure sample of fibrous phosphorus featuring parallel chains of P_8_ building blocks^10^ has been isolated by heating ultra-pure red phosphorus in the presence of CuCl_2_ in vacuo^11^. Supramolecular chemistry can promote controllable polymerisation and stabilisation of phosphorus chains within the nanochannels of a porous host. Thus, CuI has been used to stabilise phosphorus nanorods in the form of (CuI)_8_P_12_ and (CuI)_3_P_12_ adducts^12^, as well as confining and stabilising anionic P_12_^2–^ and P_14_^2–^ species^13^. Activated carbons and carbon nanotubes can adsorb tetrahedral P_4_ molecules^14^, with the latter also able to act as a host for the polymerisation of encapsulated white phosphorus molecules into single-stranded chains consisting of butterfly P_4_ fragments^15–17^. Due to the amorphous nature of these materials, their structure determination remains elusive.

A rich host–guest chemistry^18,19^, tuneable porosity, high crystallinity and potential stability of metal-organic frameworks (MOFs) make them ideal hosts for the inclusion of white phosphorus molecules, which might be further polymerised using visible light and analysed structurally by diffraction^20,21^. Moreover, the doping of MOFs with phosphorus chains of atomic width may improve the semiconducting properties of the resulting P–MOF composites. Here, we report the synthesis of a double-helical phosphorus chain comprising butterfly P_8_ monomers stabilised in the helical channels of an indium-based MOF, MFM-300(In). The similarity between Raman spectra of bulk red phosphorus and the obtained adduct suggests the presence of such helical chains within the structure of amorphous red phosphorus, thus representing a comprehensive crystallographic study on the structural building units of the red phosphorus allotrope.

## Results

### Synthesis of P_4_@MFM-300(In)

**Fig. 1:**
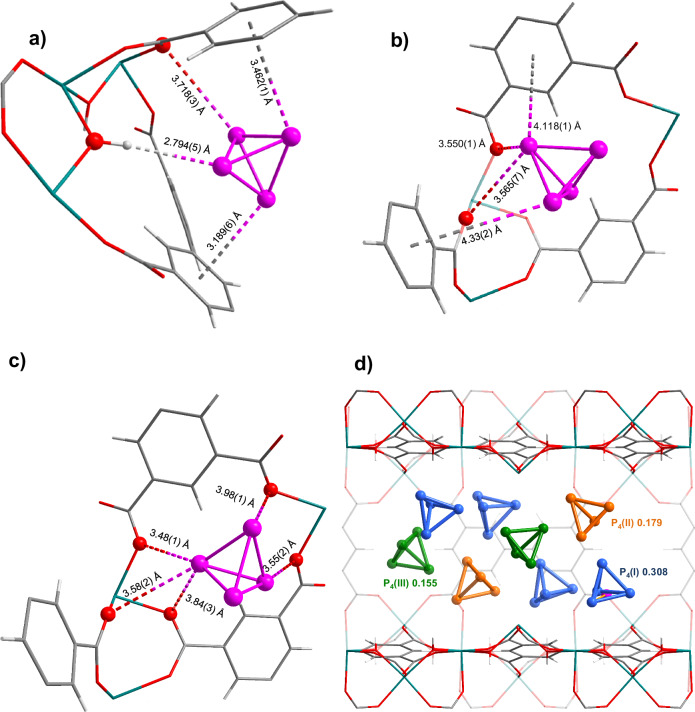
Views of the structure of P_4_@MFM-300(In) and binding modes of P_4_ in P_4_@MFM-300(In). **a** Site P_4_(I); **b** Site P_4_(II); **c** Site P_4_(III). The shortest contacts are marked with dashed lines. Indium atoms are shown green, C – grey, O – red, P – pink, H – pale grey; **d** Packing of P_4_ molecules within the channels of MFM-300(In): view in ($\bar{1}$10) plane. P_4_(I) molecules are shown blue, P_4_(II) – orange, P_4_(III) – green. The numbers indicate crystallographic occupancy.

**Fig. 2:**
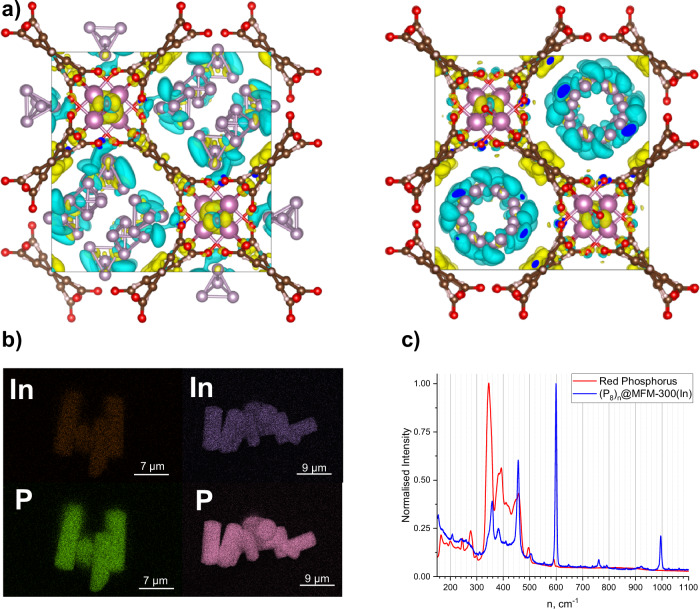
Comparative physicochemical characterisation of P_4_- and photopolymerised (P_8_)_n_@MFM-300(In). **a** Charge-density isosurfaces for the interaction of MFM-300(In) framework with (left) P_4_ molecules; (right) double helices of (P_8_)_n_. Yellow represents the accumulation and blue the depletion of the electron density. Indium atoms are shown pink, C – brown, O – red, P – pale violet, H – pale brown. The DFT results are visualised using the VESTA code; **b** EDX mapping images of In and P in P_4_@MFM-300(In) (left) and (P_8_)_n_@MFM-300(In) (right); **c** comparison between Raman spectra of commercial red phosphorus (red), and (P_8_)_n_@MFM-300(In) (blue).

MFM-300(In), [In_2_(OH)_2_(bptc)] (bptc^4–^ = 3,3′,5,5′-biphenyltetracarboxylate)^22^, was chosen for the polymerisation of white phosphorus molecules as it features non-intersecting cylindrical channels of ca. 8 Å in diameter. These are ideal to accommodate the white phosphorus P_4_ precursor and resulting polymer. Additionally, MFM-300(In) crystals are chemically stable and colourless to enable visible light-induced polymerisation. The adsorption of white phosphorus into activated MFM-300(In) was performed in the dark. Single crystal X-ray diffraction (SCXRD) analysis confirms that P_4_@MFM-300(In) crystallises in the tetragonal space group *I*4_1_22 with similar unit cell parameters [*a* = 15.5333(1) Å, *c* = 12.3260(1) Å, *V* = 2974.06(4) Å^3^] to the pristine framework. The channels are filled with tetrahedral P_4_ molecules (Fig. 1), which occupy one of three crystallographically independent sites, denoted P_4_(I), P_4_(II) and P_4_(III). The P–P distances lie in the range 2.1959(1) – 2.2277(1) Å for P_4_(I), 2.1956(1) – 2.2276(1) Å for P_4_(II), and 2.1954(1) – 2.2274(1) Å for P_4_(III), consistent with those reported for single P–P bonds^18^. Each site has a distinct binding mode. P_4_(I) is aligned towards the bridging OH-group within the channel with a O–H…P hydrogen bonding distance of 2.794(5) Å. The P_4_ molecule is favourably positioned between two phenyl rings of the ligands with close P…π-C_6_H_6_ contacts of 3.189(6) and 3.462(1) Å. There is also an interaction between P and the carboxylate oxygen atoms O_COO_ [P…O_COO_ = 3.718(3) Å] of the host. P_4_(II) molecules and their disordered counterparts P_4_(III) reside towards the centre of the pore. The shortest contacts between P_4_(II) and the framework are from the interaction of P with two carboxylic oxygen atoms O_COO_ [P…O_COO_ = 3.565(7), 3.550(1) Å]. The two phenyl rings are located further from the P atoms with P…π-C_6_H_6_ distances of 4.118(1) and 4.33(2) Å. P_4_(III) molecules show multiple interactions with the carboxylate groups [P…O_COO_ = 3.48(1), 3.55(2), 3.58(2), 3.84(3), 3.98(1) Å]. Due to translational symmetry, all of the P_4_ sites (I–III) result in a variety of possible positions of P_4_ within the channels of MFM-300(In) (Fig. 1b and Supplementary Fig. 3). Elemental analysis and TGA data (Supplementary Fig. 2) confirm the total concentration of P_4_ molecules corresponds to the formula [In_2_(OH)_2_(bptc)]·2.5P_4_·H_2_O, which also matches the structural data. The phase purity of the bulk sample was confirmed by powder X-ray diffraction (PXRD, Supplementary Fig. 5). Scanning electron microscopy (SEM) coupled with energy dispersive X-ray (EDX) mapping confirmed a homogenous distribution of phosphorus within the crystals and an absence of aggregation of phosphorus particles on the surface of the crystals (Fig. 2c and Supplementary Fig. 13). Interestingly, in contrast to white phosphorus, P_4_@MFM-300(In) is comparatively easy to handle as it does not self-ignite in air.

Periodic DFT calculations using the Vienna Ab Initio Simulation Package (VASP), with correction for Van der Waals exchange-correlation error, were performed to gain further insights into the structure of these materials^23,24^. The optimised positions of the P_4_ molecules were found to be in agreement with the crystal structure, and all the resulting adducts were found to be energetically favourable. The calculations confirm that at low concentrations of P_4_ (up to 1 P_4_ molecule per In centre), P_4_(I) is the preferred adsorption site (Supplementary Fig. 8), and at high loadings, adsorption at sites P_4_(II) and P_4_(III) occurs. The saturated material with a loading of 2 P_4_ molecules per formula unit is energetically favourable (total energy *E* = –23.6836 eV) (Supplementary Fig. 8a) and corresponds to the experimental uptake. In addition, charge distribution calculations indicate a depletion of electron density on the guest phosphorus molecules. The partial charge of the phosphorus atoms, P^δ+^, (see SI Section 5.3 and Source data file), rises as high as +0.11*e*, indicating a charge transfer between P_4_ and the framework via strong host–guest interactions (Fig. 2a).

### Conversion of P_4_@MFM-300(In) to (P_8_)_n_@MFM-300(In)

**Fig. 3:**
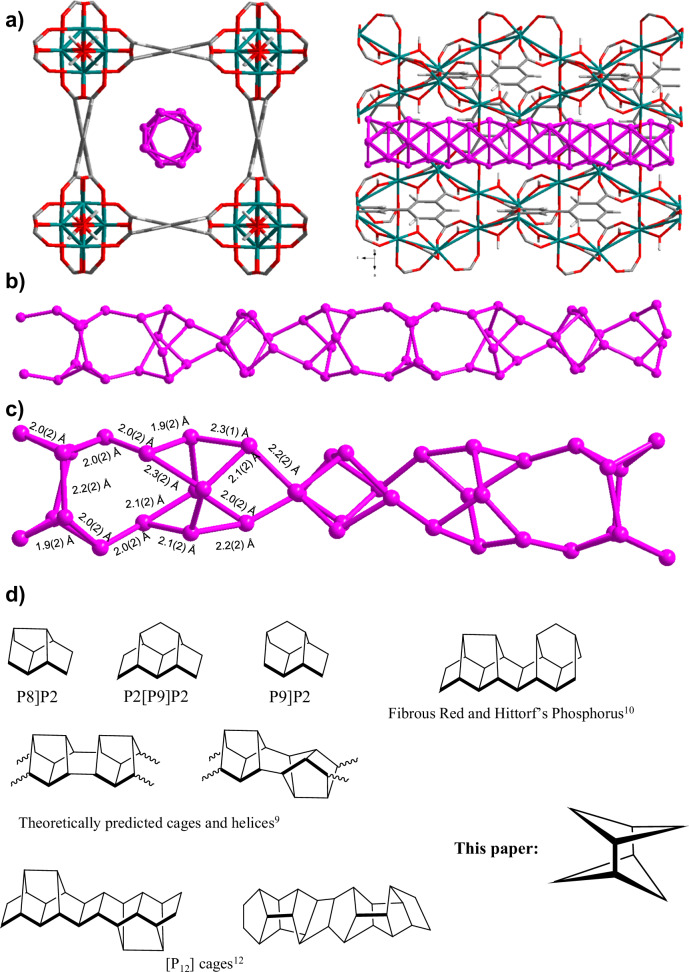
Structural studies of (P_8_)_n_@MFM-300(In) adducts. **a** The packing of the disordered nanotube of phosphorus atoms within the channels of MFM-300(In): view along *c* axis (left*)* and in ($\bar{1}$10) plane (right). Indium atoms are shown green, C – grey, O – red, P – pink, H – pale grey; **b** View of the DFT-optimised double helix; **c**) view of the fragment of the double helix refined from powder diffraction data; **d** Comparison of P_8_ unit with other P_n_ cluster fragments predicted or experimentally observed in polymeric phosphorus allotropes.

P_4_@MFM-300(In) was irradiated with a 300 W Xe light source (λ = 350–760 nm) to induce the polymerisation of the P_4_ species within the channels. Within 6–8 h (depending on the amount of starting material), the pale yellow crystals turned deep orange with full retention of crystallinity (Supplementary Figs. 6, 7). SEM and EDX show a homogenous distribution of phosphorus within the samples, with full retention of morphology and the absence of phosphorus particles on the external surfaces after the polymerisation (Fig. 2c). Elemental analysis confirms a concentration of phosphorus of 30 wt% in the polymerised adduct, comparable with the initial concentration of phosphorus in P_4_@MFM-300(In) (34 wt%). N_2_ adsorption measurements at 77 K confirm full occupation of the pore space in (P_8_)_n_@MFM-300(In) since it is not porous to N_2_. This is in contrast to bare MFM-300(In), which shows a BET surface area of 1000 m^2^ g^–1^ (see SI Section 10). SCXRD analysis confirms the integrity of the host framework with no apparent structural change in the MOF platform. In contrast, the structure of guest phosphorus changes significantly on irradiation, with four crystallographically independent phosphorus atoms forming connected butterfly P_4_ fragments of C_2v_ symmetry. These are similar to the P_4_ fragments that have been observed in coordination and organophosphorus compounds^25,26^. The distribution of P–P bond distances is slightly distorted in the fragment, and ranges from 1.934(5) to 2.243(5) Å. The fragments are aggregated into a chain of elemental phosphorus running along the *c*-axis and can be viewed as connected P_8_ clusters (Fig. 3a and Supplementary Fig. 5). The same tubular structure was observed independently by refining high-resolution PXRD data from the powder sample of (P_8_)_n_@MFM-300(In) (Supplementary Fig. 6 and Supplementary Tables 4, 5) in which the refined P_n_ moieties show a similar variation of P–P bond length [2.0(2) - 2.5(2) Å]. The P(3)P(2)P(4), P(2)P(4)P(3′) and P(3′)P(3)P2 angles are 74(2)°, 96(2)°, and 101(2)°, respectively. These P_4_ moieties are chemically bound to each other to form a unique P_8_ building block which forms a double helix chain running along the *c* axis. These cluster and double-helical chains are unusual in the structural chemistry of elemental phosphorus (Fig. 3d and Supplementary Fig. 4). Interestingly, the P–P bond lengths within helices of butterfly P_4_ subunits are similar [1.99(5) – 2.01(5) Å], while the P–P distance between two P_4_ subunits in the P_8_ building block is substantially longer [2.24(5) Å, See Supplementary Fig. 5]. This latter value is comparable with P–P bond length reported for sterically hindered diphosphanes [2.291(4)–2.357(2) Å]^27^ and in hypothetical small phosphorus cluster molecules [2.24 Å for one of possible P_6_ clusters]^28^, indicating weaker bonding. To confirm that the observed double helix is not a mere result of the structural disorder of P_4_ subunits and to explain the deviation in P–P–P angles, we used ab initio DFT calculations to analyse the system (See Methods and SI Section 5 for details). The analysis confirms that single chains of butterfly P_4_ fragments, which have been thought to be the main components of red phosphorus since the 1950s^29^, collapse within the channels of the host MOF framework. The resultant double helix [P_8_]_n_ polymer (Fig. 3b and Supplementary Figs. 10, 11) is thermodynamically stable with a total energy for its optimised form of –16.62718 eV (–0.51960 eV per phosphorus atom).

A distortion in each of the P_4_ subunits within the cluster can be rationalised by considering the redistribution of electron density and charge transfer between the double helix and the framework. DFT calculations performed on the optimised (P_8_)_n_@MFM-300(In) fragment reveal a depletion of electron density on the phosphorus atoms and charge transfer between the phosphorus chain and the functional groups of the host framework (Fig. 2a). The partial charges can reach +0.092*e* (see SI Section 5.3 and Source data file). The depletion of the electronic density in the P_4_ subunit, as well as steric hindrance, explains the flattening of the fragment in comparison to known neutral organophosphorus compounds featuring the same fragment. Indeed, it has been shown previously that electron depletion can cause a flattening of the P_4_ fragments, for example, in the cationic form {(Me_2_P^+^)_2_(*t*-Bu_2_P)_2_} where the P_4_ cluster is significantly flattened with a fold angle for the P_4_ scaffold of 139.4°^30^. Binding to metal centres and emergence of ligand to metal charge transfer, especially within a sterically hindered geometry, may lead not only to flattening, but also to aromatisation within a cyclic P_4_ moiety^31–34^.

### Spectroscopy

The formation of the double helix chain has also been tracked by Raman spectroscopy. The Raman spectrum of P_4_@MFM-300(In) features three intense peaks at 361, 467, and 601 cm^–1^ corresponding to Raman-active *F*_2_, *E*, *A*_1_ vibrations of the P_4_ tetrahedron, respectively (Supplementary Fig. 14a)^35^. On polymerisation, new peaks in the Raman spectrum appear at 357(m), 381(m), 409(w), 456(s), 501(w), and 599(s) cm^–1^ (Fig. 2c). These match the corresponding peaks of amorphous red phosphorus at 345(s), 382(m), 410(w), 458(m), 496(w), and 591(w) cm^–1^ (Fig. 2c), and suggest the presence of such helical chains in red phosphorus. It is worth noting that both spectra are very different from the predicted Raman spectra of any single chain built up from P_4_ fragments^36^. Thus, the obtained double helix structure observed here is consistent with one of the important components of amorphous red phosphorus. This is distinct from a predicted molecular structure of amorphous red phosphorus^37^ and experimentally observed double helices within carbon nanotubes^38^. The similarity between red phosphorus and photopolymerised species in (P_8_)_n_@MFM-300(In) can be observed by NMR spectroscopy as well (See SI Section 8). The solid-state ^31^P NMR spectrum of (P_8_)_n_@MFM-300(In) shows one broad signal at –120 ppm, which is close to one of the peaks observed in the NMR spectrum of pure red phosphorus (Supplementary Fig. 15). The Raman spectra of freshly prepared and 3-month-old (P_8_)_n_@MFM-300(In) demonstrate no significant differences (Supplementary Fig. 14b), confirming good general stability of the material toward prolonged exposure to air. A weak signal at 924 cm^−1^ is present in the spectra of fresh and air-exposed samples and is characteristic of the presence of P–O bonds^39^. However, the consistently low intensity of this peak in both spectra suggests that this represents only surface oxidation.

To gain further understanding of the electronic structure of these compounds, we determined the bandgap of MFM-300(In), P_4_@MFM-300(In) and (P_8_)_n_@MFM-300(In) by UV-Vis spectroscopic experiments (see SI Section 9). Upon adsorption of P_4_ and its polymerisation, the bandgap decreases from 3.9 in MFM-300(In) to 3.2 in P_4_@MFM-300(In) to 2.3 eV in (P_8_)_n_@MFM-300(In) (Table S6). This is a narrower bandgap than in most guest-free MOF complexes such as MIL-101, ZIF-8, HKUST-1 and MOF-74, and is comparable with other well-performing semiconducting MOF-based host–guest systems (Table S7). The decrease in bandgap in going from MFM-300(In) to P_4_@MFM-300(In) to (P_8_)_n_@MFM-300(In) is explained by partial density of states (PDOS) calculations (Supplementary Fig. 12). In short, the narrowing of the bandgap in this system is caused by the contribution of phosphorus orbitals to the valence band maximum (VBM) and the conduction band minimum (CBM), which is the largest in the case of (P_8_)_n_@MFM-300(In). In guest-free MFM-300(In), the VBM and CBM are affected primarily by the orbitals on the bridging ligand.

The decrease in bandgap in the composite (P_8_)_n_@MFM-300(In) affects a number of physical properties compared to the bare MOF, allowing SO_2_ detection by monitoring changes in the dielectric constant of the material (see SI Section 11.1). Importantly, the phosphorus adduct has a much higher ability to generate a photo-current (SI Section 11.3, Supplementary Fig. 22), and is luminescent (SI Section 12, Supplementary Fig. 23), key features for photo-catalysis and for water purification^40^. To examine this effect, guest-free MFM-300(In) and (P_8_)_n_@MFM-300(In) were tested for the photocatalytic degradation of rhodamine B and methyl orange as model compounds of water pollutants. In each test, the concentration of the catalyst and the pollutant was 0.5 and 0.01 g L^–1^, respectively. No additional oxidant other than air was used. Prior to irradiation with an incandescent halogen lamp (250 W), the catalyst was dispersed in the solution for 30 min to reach adsorption equilibrium. The reaction rate was monitored by the UV-Vis spectroscopy (Supplementary Figs. 24, 25). As expected, the inclusion of the phosphorus chain within MFM-300(In) boosts the catalytic performance. Thus, (P_8_)_n_@MFM-300(In) promotes the oxidation of Rhodamine B with a 90% conversion of a substrate after 150 min of irradiation, while the reaction with MFM-300(In) shows a conversion of just 56% after 270 min. The use of red phosphorus resulted in only 10.6% conversion after 150 min. Moreover, bare MFM-300(In) catalyses the oxidation of methyl orange poorly, with a conversion of only 9.6% after 300 min, whereas (P_8_)_n_@MFM-300(In) demonstrates a tenfold increase in conversion to 90.3% in 300 min. Bulk red phosphorus is also very efficient in this reaction, demonstrating 99% conversion after 300 min. The heterogenous nature of catalysis was confirmed by filtration tests (Supplementary Fig. 26), in which the solid catalyst is removed and subsequent catalysis monitored. The stability of the catalyst was monitored and confirmed by PXRD measurements (Supplementary Fig. 27). Overall, the introduction of polymeric phosphorus significantly improves the photocatalytic performance of the resulting material, showing superior catalytic performance to MOF materials such as UiO66^41^, MIL-53^42^, MIL-100^43^, and bismuth oxide-MOF adducts^44^. High conversion rates are often reported when hydrogen peroxide is used as an oxidant (Table 1). However, the use of hydrogen peroxide has several disadvantages compared to air, as H_2_O_2_ is an aggressive oxidant and may cause explosions and equipment failures when used in industrial scale^45^. The conversion of Rhodamine B using (P_8_)_n_@MFM-300(In) is especially effective, and a prepared sample of milled red phosphorus^46^ was able to reach 88% conversion of Rhodamine B, but in double the length of time compared with (P_8_)_n_@MFM-300(In). Table 1 summarises data on the photo-degradation of dyes using for (P_8_)_n_@MFM-300(In) and other MOFs and P-containing materials.

**Table 1:** Selected examples of photocatalytic degradation of dyes by MOFs and P-containing materials

Catalyst	Catalyst concentration g/L	Dye*	Oxidant	Time	Conversion %	
P_8_-MFM-300	0.5	10 mg/L RhB	Air	150 min	90	This work
P_8_-MFM-300	0.5	10 mg/L MO	Air	300 min	90	This work
Milled red phosphorus (RP-36)	0.4	10 mg/L RhB	Air	6 hours	88	^46^
Bulk black phosphorus	1.0	2·10^-4^ mg/L MO	Air	120 min	25	^62^
Black phosphorus nanosheets @ Graphene Oxide	1.0	2·10^-4^ mg/L MO	Air	120 min	100	^62^
nMLM	0.1	50 mg/L RhB	1 ml H_2_O_2_	240 min	92	^63^
MIL-53-Fe	0.01	4·10^-4^ M MB	Air	1 h	11	^42^
UiO66	0.1	20 mg/L MO	Air		0	^41^
UiO66-1.25Ti	1.0	10 mg/L MB	Air	80 min	82	^64^
HKUST-1	0.125	25.28 mg/L RB13	Air	14 h	40	^65^
MIL-100 (Fe)	0.3	5 ppm MO	Air	7 h	64	^43^
Au@MIL-100(Fe)	0.125	0.02 g/L MO	10 mM H_2_O_2_	1.57 h	100	^66^
(Fe-Ti)-(MOF-NH2) (3:1)	0.1	0.05 g/L OrangeII	14 mM H_2_O_2_	10 min	100	^66^
(Fe_3_O_4_)-(MIL-88B (Fe))	0.2	MB/ RhB 0.1:0.01 mM	20 mM H_2_O_2_	1.33 h	100	^66^
MIL88(Fe)	0.4	10 mg/L RhB	20 mM H_2_O_2_	80 min	50	^67^
Bi_2_O_3_ – Cu-MOF	1.0	10 mg/L RhB	Air	60 min	44	^44^
Bi_2_O_3_ – Cu-MOF– Graphene Oxide (BCG-2)	1.0	10 mg/L RhB	Air	60 min	80	^44^

^*^RhB rhodamine B, *MO* methyl orange, *MB* methylene blue

## Discussion

In summary, the encapsulation and photo-polymerisation of guest white phosphorus molecules in P_4_@MFM-300(In) have been investigated. The high crystallinity of the adduct allowed the determination of the structure of P_4_@MFM-300(In) and of the chain allotrope (P_8_)_n_@MFM-300(In), the latter demonstrating an unusual helical shape stabilised within the pores of the MOF. Raman spectroscopy confirms that the helical phosphorus chain observed in (P_8_)_n_@MFM-300(In) shows similar characteristics to that of amorphous red phosphorus. The confinement of phosphorus chains in MFM-300(In) significantly changes its functional properties, and the resultant composite is an air-stable semiconductor with a bandgap of 2.3 eV, with potential for gas detection and photocatalytic applications. This methodology to stabilise otherwise unstable species within a porous host can potentially be extended to other porous OH-decorated MOFs, since P_4_ molecules strongly interact with hydroxyl groups, as demonstrated here. Other small molecules based upon S, Se, As and Sb might also be used as substrates for MOF-assisted photo-polymerisation, which will be a powerful way to modify and control the electronic structure and photocatalytic performance of the resulting encapsulated and protected adducts, aggregates and polymers.

## Methods

### Materials and characterisation techniques

Indium(III) nitrate hydrate (Aldrich, 99.9%), biphenyl-3,3′,5,5′-tetracarboxylic acid (TCI, 98%), DMF (Fisher Chemical, 99.8%), acetonitrile (Fisher Chemical, 99.5%), concentrated nitric acid (Fisher Chemical, 67–70 wt%), red phosphorus (Aldrich, 97%), Rhodamine B (Fisher Chemical, 95%), methyl orange (Aldrich, 95%), sodium sulphate (Aldrich, 99.0%), Nafion D-521 (Fisher Chemical, 5 wt% dispersion in water and 1-propanol, 0.92 meq g^–1^ exchange capacity) were used as purchased. White phosphorus was obtained from red phosphorus^47^. Elemental analysis was performed on a Flash 2000 elemental analyser, and the morphology of the crystallites was determined on a SEM on a Quanta 650 microscope. A Shimadzu UV-2600 spectrometer was used to collect solid-state UV-Vis spectra in the range from 800 to 200 nm, and solid-state luminescence excitation and emission spectra recorded on an Edinburgh Instrument FP920. Solid-state ^31^P NMR spectra were collected on a Bruker AVIII 400 instrument, and TGA plots (Supplementary Fig. 2) were collected on a Perkin Elmer Pyris1 thermogravimetric analyser under N_2_ at a flow rate of 100 mL/min and heating rate of 5 °C /min. Raman spectra were collected on a Horiba Scientific Xplora plus Raman Microscope using a 785 nm laser with a grating of 1200 gr mm^–1^. A total of 10 acquisitions with an exposure time of 10 s were used to collect spectra. A 300 W Xe lamp was used at a wavelength of 350–760 nm (visible output 5000 lm) for irradiation of P_4_@MFM-300.

### Synthesis of MFM-300(In)

MFM-300(In) was obtained according to a modification of the published method^22^ as follows: a mixture of In(NO_3_)_3_·5H_2_O (0.390 g, 1.0 mmol), biphenyl-3,3′,5,5′-tetracarboxylic acid H_4_L (0.033 g, 0.1 mmol), DMF (10 ml) and acetonitrile (10 ml) was acidified with 60 drops of concentrated HNO_3_. The resulting slurry was sealed in a pressure flask, sonicated in an ultrasonic bath and heated at 80 °C for 3 days. This yielded a highly crystalline material, which was filtered-off, washed five times with DMF and dried on air. The sample was activated by soaking in acetone for a week with subsequent heating in a dynamic vacuum at 120 °C overnight. Yield 0.041 g (70%, based on H_4_L).

### Synthesis of P_4_@MFM-300(In)

CAUTION: White phosphorus is highly toxic and self-ignites in air. All the operations with white phosphorus must be performed under an  inert atmosphere using the Schlenk technique. Due to the light-sensitivity of the target compound and of P_4_, the reaction should be carried in darkness.

Activated guest-free MFM-300(In) powder was placed in a Schlenk vessel, which was connected to another Schlenk flask filled with white phosphorus (Supplementary Fig. 1). The setup was evacuated, the valves closed (without refilling the setup with inert gas), and the flask with the white phosphorus was heated on a water bath for 1 h. The setup was cooled and left at room temperature for 3 days in darkness to ensure maximum adsorption of phosphorus vapour by MFM-300(In). To remove excess condensed white phosphorus, the flask with MFM-300(In) was disconnected and connected to a clean receiving Schlenk flask. The setup was degassed again, and the flask with MFM-300(In) was heated in the water bath at 50 °C for 1–2 h, then cooled down and left overnight. The setup was refilled with N_2_, and the receiving flask containing excess white phosphorus was disconnected. The obtained pale-yellow powder of P_4_@MFM-300(In) was stored in the Schlenk vessel under an inert atmosphere in the dark (in a box or fully wrapped with tin foil) as the sample is light-sensitive even to normal daylight. Analysis (calcd., found for In_2_C_16_H_10_O_11_P_10_): C (20.54, 20.94), H (1.29, 1.10), N (0, 0), P (33.10, 33.75). TGA (Δ*m*, %): –2% (H_2_O), –34% (2.5P_4_).

### Synthesis of (P_8_)_n_@MFM-300(In)

A sample of P_4_@MFM-300(In) (0.100 g, 0.109 mmol) was placed in a Petry dish and irradiated with a 300 W Xe lamp until all the samples turned deep orange. The powder was stirred every 2 h to ensure that all the sample was irradiated fully. The irradiation time depends on the amount of starting material but usually takes 6–8 h. The obtained orange material was transferred into a Schlenk vessel and heated in a dynamic vacuum overnight at 100 °C. Yield 0.098 g (90%). Analysis (calcd., found for In_2_C_16_H_8_O_10_P_9,7_(H_2_O)_5_): C (19.60, 19.75), H (1.85, 1.40), N (0, 0), P (30.65, 30.38). TGA (Δ*m*, %): –9% (5H_2_O).

### Details of DFT calculations

First-principles calculations were performed within the framework of density functional theory (DFT), as implemented in the Vienna Ab initio Simulation Package (VASP version 5.4.4)^23,24^. Electron exchange-correlation was treated by the generalised gradient approximation (GGA) with Perdew, Burke, and Ernzerhof (PBE) parameterisation^48^, and interactions between the ion cores and valence electrons were modelled by the all-electron projector augmented wave (PAW) method^49,50^. The plain-wave cutoff energy was 400 eV, and convergence in energy (10^–4^ eV) and force (3 × 10^–3^ eV/Å) were used during the optimisation procedure. Brillouin zone integrations were performed using the Monkhorst-Pack k-point mesh^51^ with the 4 × 4 × 5 and 2 × 2 × 1 grids for calculation of single cell and 1 × 1 × 3 supercell, respectively. In order to properly estimate the weak non-covalent interactions, the Grimme parameterisation was applied^52^.

The adsorption energies (*E*_ads_) were calculated as the difference between the sum of the binding energies of the empty MOF (*E*_host_) and the number of non-coordinated guest P_4_ clusters (*n*·*E*_guest_), where *n* is the number of adsorbed non-coordinated P_4_ clusters, and that of the adsorbed system (*E*_host+guest_) and the adsorption state, with a negative value of *E*_ads_ being thermodynamically favourable [Eq. (1)].

1 $${E}_{{ads}}={{E}_{host+guest}}{{{\rm{\hbox{-}}}}}({E}_{{host}}+n\, {{\cdot }}\,{E}_{{guest}})$$

In the case of chain calculations, *E*_guest_ was considered for a single chain configuration. The interaction between the porous host and the substrate has been demonstrated by a charge-density isosurface, and the difference in charge density (excess and depletion electrons) was estimated as:

2 $$\Delta {{{\rm{\rho }}}}={{{\rm{\rho }}}}\left({host}+{guest}\right)-{{{\rm{\rho }}}}\left({host}\right)-{\sum }_{k=1}^{n}{{{{\rm{\rho }}}}}_{k}\left({guest}\right)$$

and the obtained charge density isosurfaces for the guest−host interactions were visualised using the VESTA code^53^.

The theoretical analysis described above has been verified in previous studies^54–57^. The effective charge of atoms was evaluated by using the Bader analysis algorithm^58–61^.

### Mott-Schottky plot and photo-current measurements

Mott-Schottky plots were recorded on a CHI660E workstation (CH Instruments, USA) with a conventional three-electrode system using a 0.5 M Na_2_SO_4_ aqueous solution. Preparation of the working electrode: 4 mg of bulk red phosphorus, MFM-300(In) or (P_8_)_n_@MFM-300(In) were dispersed in a solution of 4 mL ethanol and 10 μL Nafion D-521 to generate a homogeneous slurry. About 10 μL of the slurry was transferred and coated onto glassy carbon (diameter of 3 mm) and then dried. An Ag/AgCl electrode was employed as the reference electrode, and a platinum plate was used as the counter electrode.

### Photocatalytic experiments

About 5.0 mg of the catalyst [(P_8_)_n_@MFM-300(In), guest-free MFM-300(In) or red phosphorus] was dispersed in 10 mL of the 0.1 g L^−1^ aqueous solution of rhodamine B or methyl orange. After vigorous stirring in the dark for 30 min, the reaction mixture was irradiated with an incandescent 250 W halogen lamp for 300 min. The rate of the reaction was monitored by recording the UV-Vis spectra of the aliquots taken 30, 90, 150, 210, and 300 min after the beginning of the reaction. After completion of the reaction, the solid-state catalyst was separated by centrifugation at 11872 x *g* for 15 min, washed multiple times with acetone and dried on air. For the filtration test, the catalyst was removed by filtration after 30 mins of irradiation and by centrifugation at 11,872×*g* for 15 min. The remaining liquid phase was further irradiated for 270 min and analysed by UV-Vis spectroscopy. For blank tests, the catalyst and the dye solution taken in the same ratio as described above were stirred in complete darkness for 24 h. The concentration of dye in these solutions was monitored by UV-Vis spectroscopy.

## Supplementary information

Supplementary Information

Transparent Peer Review file

## Source data

Source Data

## Data Availability

TGA, single-crystal, powder X-ray and synchrotron diffraction analyses, details of DFT calculations, SEM data, Raman, SNMR, UV-Vis spectroscopy data, adsorption isotherms, electrochemical, photo-luminescence, and photocatalytic experimental data generated in this study in the Supplementary Information. The crystallographic data of the materials reported in this work have been deposited in the Cambridge Crystallographic Data Centre (CCDC) under accession numbers CCDC 2255227 and 2255484 for the single-crystal structures of P_4_@MFM-300(In) and(P_8_)_n_@MFM-300(In), respectively, and 2255485 for the structure of (P_8_)_n_@MFM-300(In) refined by the Rietveld Method. All data were available from the corresponding authors upon request. Source data are provided with this paper.
