# Supplementary material for: Direct synthesis of a semiconductive double-helical phosphorus allotrope in a metal-organic framework
Source: Nat Commun. 2025 Feb 12;16:1578. doi: 10.1038/s41467-025-55999-4 (PMC11821825; doi:10.1038/s41467-025-55999-4)
Supplement: Supplementary file 1 — Supplementary Information [file 41467_2025_55999_MOESM1_ESM.pdf]

Electronic Supplementary Information

Direct synthesis of a semiconductive double-helical phosphorus allotrope in a metal-organic framework

Sergei A. Sapchenko,[1]* Rodion V. Belosludov,[2] Inigo J. Vitoria-Irezabal,[1] Ivan da Silva,[3] Xi Chen,[1,4] George F.S. Whitehead,[1] John Maddock,[1] Louise S. Natrajan,[1] Meredydd Kippax-Jones,[1] Dukula De Alwis Jayasinghe[1], Carlo Bawn,[1] Daniil M. Polyukhov,[1] Yinlin Chen,[1] Vladimir P. Fedin,[5,6] Sihai Yang,[1,7]* and Martin Schröder[1]*

[1] Department of Chemistry, University of Manchester, Manchester M13 9PL (UK)

[2] Institute for Materials Research, Tohoku University, Sendai 980-8577 (Japan)

[3] ISIS Facility, STFC Rutherford Appleton Laboratory, Oxfordshire OX11 0QX (UK)

[4] College of Chemistry and Chemical Engineering, China University of Petroleum (East China), Qingdao 266580 (PR China)

[5] Nikolaev Institute of Inorganic Chemistry SB RAS, 3 Lavrentiev Ave., Novosibirsk 630090 (Russian Federation)

[6] Faculty of Natural Sciences, Novosibirsk State University, 1 Pirogov Str., Novosibirsk 630090 (Russian Federation)

[7] College of Chemistry and Molecular Engineering, Beijing National Laboratory for Molecular Sciences, Peking University, Beijing 100871, (PR China)

TABLE OF CONTENTS

1. Experimental Details 3

2. Thermogravimetric analysis 4

3. Single Crystal X-Ray Crystallography 5

4. Powder X-ray and Synchrotron Diffraction Analyses 8

5. Details of DFT Calculations 10

6. Scanning Electron Microscopy 15

7. Raman Spectroscopy 16

8. Solid-state NMR Spectroscopy 17

9. UV-Vis Spectroscopy and Calculation of Band Gap Energy 17

10. Nitrogen Adsorption Studies 19

11. Electrochemical Studies 20

12. Photo-luminescence Studies 24

13. Photo-catalytic Experiments 26

14. Supplementary References 28

1. Experimental Details

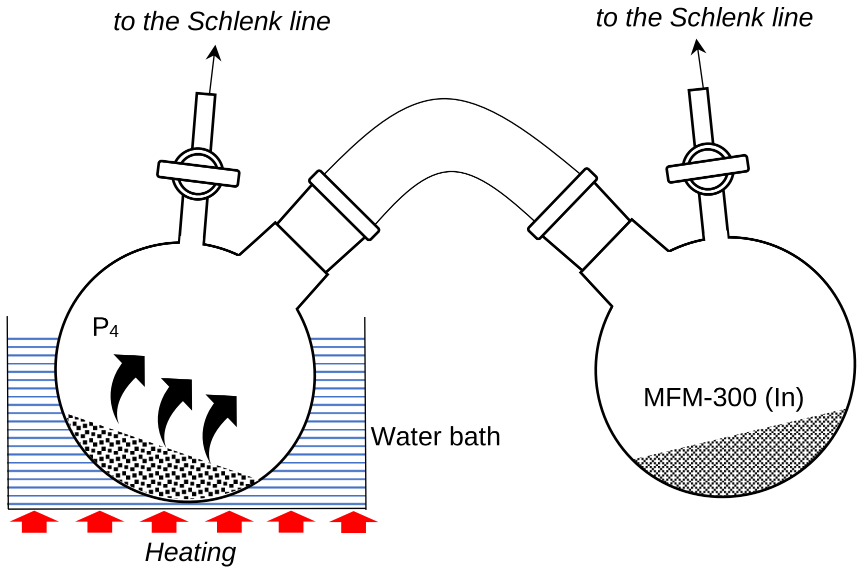

Supplementary Fig. 1. The experimental set-up for the synthesis of P_4@MFM-300(In).

2. Thermogravimetric Analysis

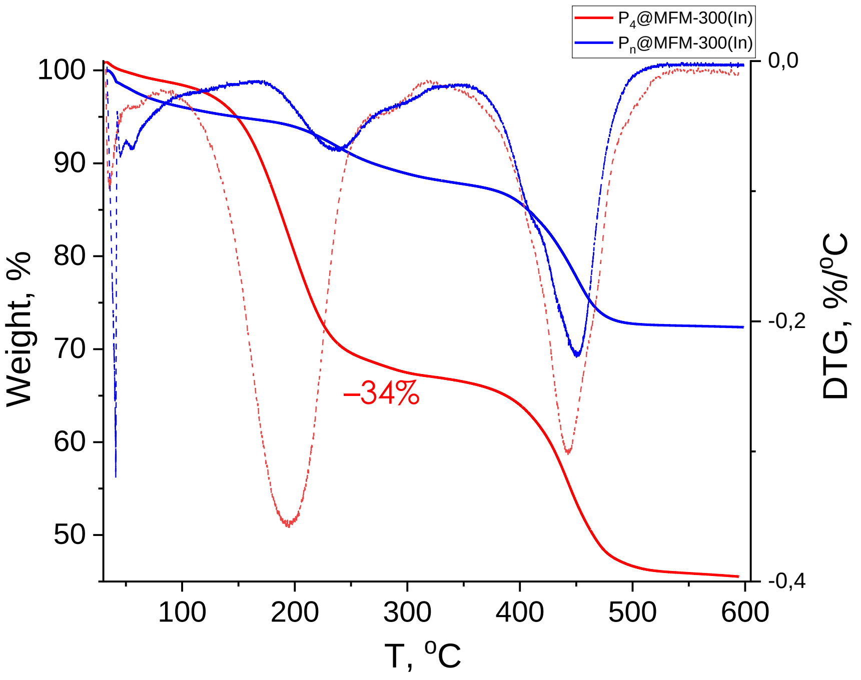

Supplementary Fig. 2. TG and DTG plots (dashed) for P_4@MFM-300(In) (red) and $(P_8)_n$@MFM-300(In) (blue) recorded at the heating rate of 5° min^{-1} in the inert atmosphere. P_4 is volatile and evaporates from the sample of P_4@MFM-300(In) upon heating. $(P_8)_n$ in $(P_8)_n$@MFM-300(In) is much less volatile and therefore the weight loss for P_4@MFM-300(In) occurs at a lower temperature.

3. Single Crystal X-Ray Crystallography

Single crystal X-ray data were collected at 150 K on a GV1000 Oxford-Rigaku Supernova diffractometer with Mo-Kα (λ = 0.71073Å) and at 100 K using Rigaku FR-X diffractometer with Cu-Kα (λ = 1.54184Å) equipped with a CCD detector and an Oxford Cryosystems liquid N_2 flow system. Data collection, frame integration and data processing were performed using CrysAlisPro program suite[1]. The structure was solved by direct method and refined on F^2 by full-matrix least-squares method in the anisotropic approximation (for non-hydrogen atoms) using Olex2 program package[2]. The positions of hydrogen atoms of organic ligands were calculated geometrically and refined by the riding model. A summary of the crystallographic data and structural determination for all the compounds is provided in Supplementary Table 1.

Supplementary Table 1. Crystal data and structure refinement for P_4@MFM-300(In) and P_n@MFM-300(In)

Identification code	**P_4@MFM-300(In)**	**$(P_8)_n$@MFM-300(In)**
Empirical formula	$C_8H_4InO_5(P_4)_{1.284}$	$C_8H_4InO_5P_4$
Molar mass, g/mol	453.97	418.81
T, K	100	100
Crystal system	Tetragonal	Tetragonal
Space group	$I4_122$	$I4_122$
a, Å	15.5333(1)	15.4523(3)
c, Å	12.3260(1)	12.3324(3)
V, Å^3	2974.06(5)	2944.7(1)
Z	8	8
D_{calcd}, g/cm^3	2.028	1.889
μ, mm^{-1}	18.067	17.051
F(000)	1744	1608
Crystal size, mm	0.01 × 0.01 × 0.03	00.1 × 0.01 × 0.05
θ range, deg	4.0 – 76.2	4.0 – 76.1
Limiting indices *hkl*	$-19 \le h \le 19$<br>$-19 \le k \le 19$<br>$-15 \le l \le 15$	$-19 \le h \le 19$<br>$-19 \le k \le 18$<br>$-15 \le l \le 15$
Reflections collected/unique	19963 / 1553	17349 / 1552
R_{int}		0.061
T_{max} / T_{min}	1.0000 / 0.6874	1.00000 / 0.58665
Goodness-of-fit (GoF) on F^2	1.096	1.17
Final *R* indices [$I > 2\sigma(I)$]	R_1 = 0.0301<br>wR_2 = 0.0807	R_1 = 0.0369<br>wR_2 = 0.1037
Largest difference in peak and hole, *e*/A^3	–0.72, 0.80	–0.74, 0.78
CCDC number	2255484	2255227

Supplementary Table 2. Selected Bond Distances and Angles in P_4@MFM-300(In)

Bond Distances			
Bond	*d*, Å	Bond	*d*, Å
In(1) – O(1)	2.1310(1)	In(1) – O(3)	2.0994(1)
P(1) – P(2)	2.2223(1)	P(1) – P(3)	2.1959(1)
P(1) – P(4)	2.1959(1)	P(2) – P(4)	2.1965(1)
P(2) – P(3)	2.2089(1)	P(3) – P(4)	2.2277(1)
Bond Angles			
Angle	ω, deg	Angle	ω, deg
P(3) – P(1) – P(4)	60.96(1)	P(2) – P(1) – P(3)	59.99(1)
P(2) – P(1) – P(4)	59.62(1)	P(1) – P(2) – P(3)	59.41(1)
P(1) – P(2) – P(4)	59.59(1)	P(3) – P(2) – P(4)	60.75(1)
P(1) – P(3) – P(2)	60.60(1)	P(2) – P(3) – P(4)	59.35(1)
P(1) – P(3) – P(4)	59.52(1)	P(1) – P(4) – P(3)	59.52(1)
P(2) – P(4) – P(3)	59.90(1)	P(1) – P(4) – P(2)	60.79(1)

Supplementary Table 3. Selected Bond Distances and Angles in P_n@MFM-300(In)

Bond Distances			
Bond	*d*, Å	Bond	*d*, Å
In(1) – O(1)	2.126(7)	In(1) – O(3)	2.099(5)
P(1) – P(2)	2.00(5)	P(2) – P(3)	2.01(5)
P(3) – P(4)	2.02(5)		
Bond Angles			
Angle	ω, deg	Angle	ω, deg
P(1) – P(2) – P(3)	101(2)	P(2) – P(3) – P(4)	99(2)

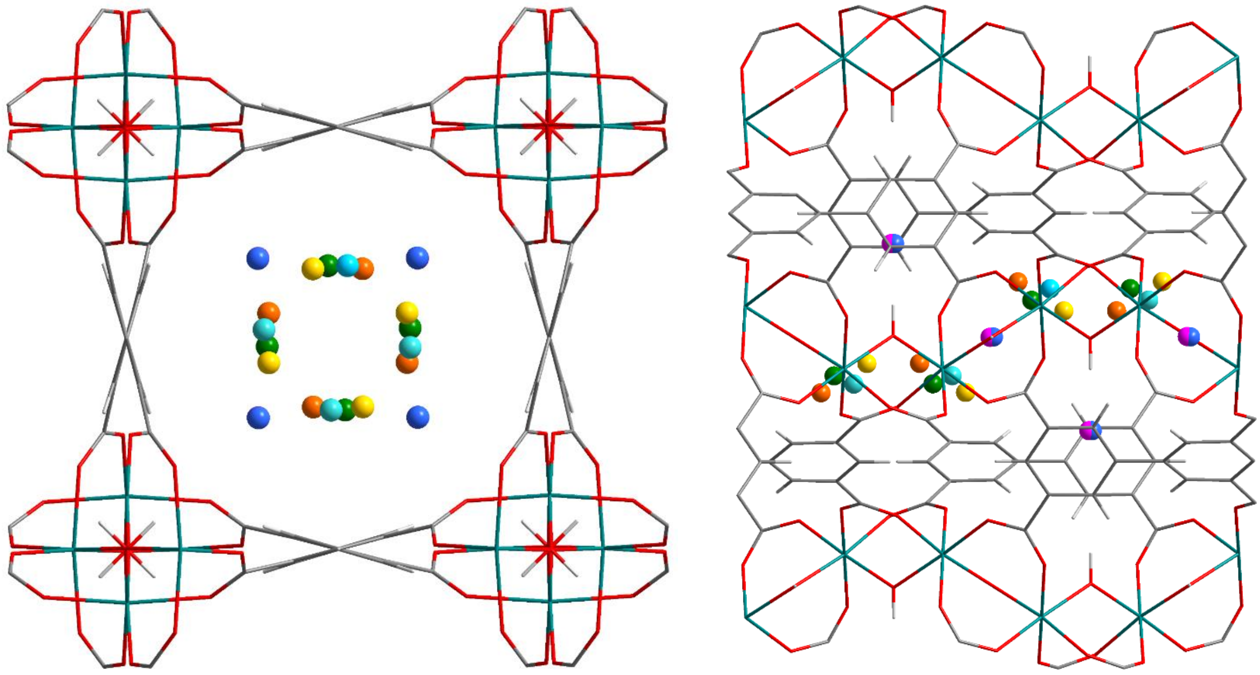

Supplementary Fig. 3. Schematic representation of all the adsorption cites within the channels of P_4@MFM-300(In). The coloured spheres correspond to the geometrical centres of P_4 molecules.

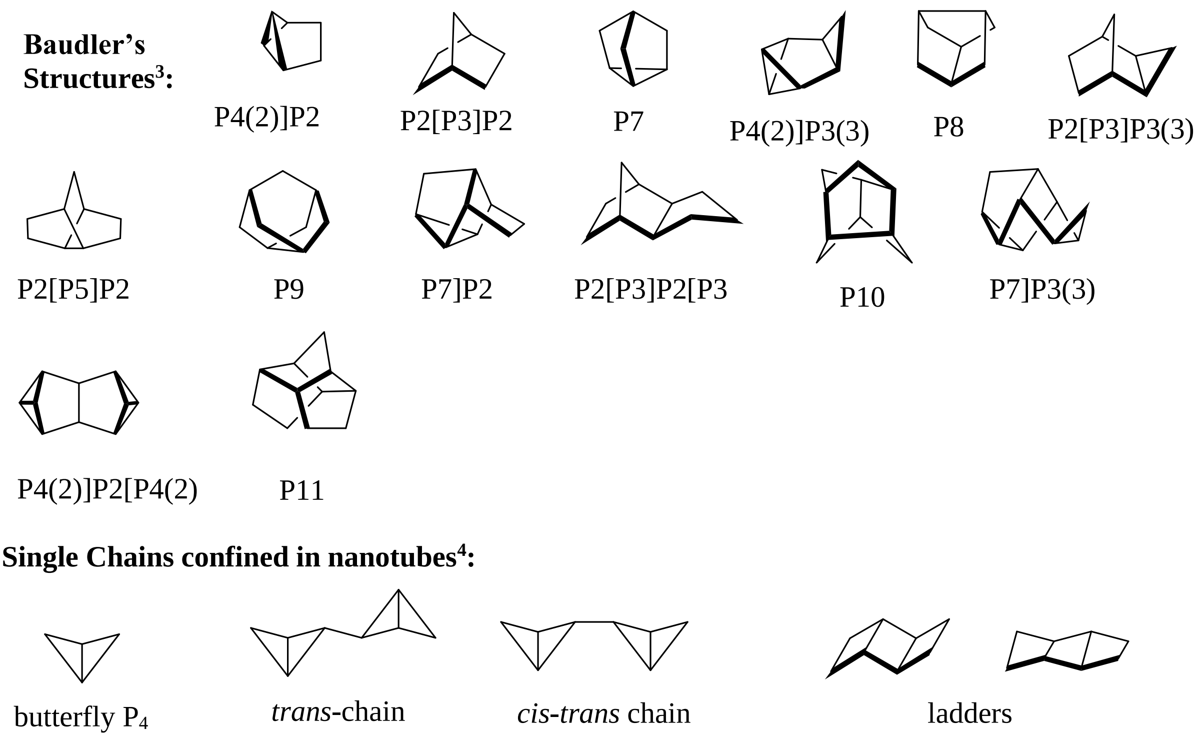

Supplementary Fig. 4. View of P_n cluster fragments predicted or experimentally observed in the polymeric P allotropes.

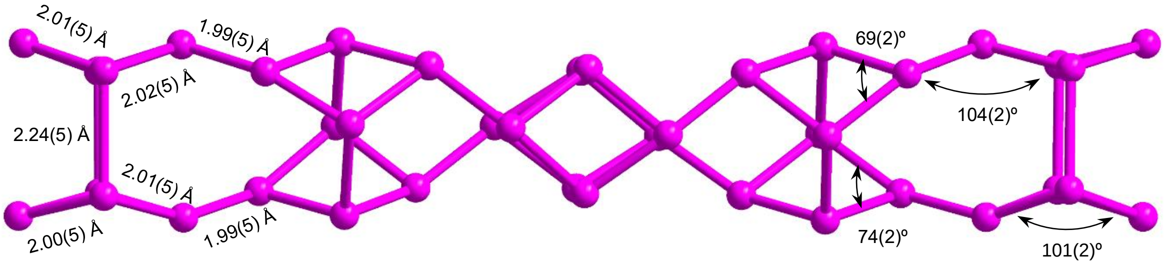

Supplementary Fig. 5. The fragment of the double helix refined from single crystal diffraction data for $(P_8)_n$MFM-300(In)

4. Powder X-ray and Synchrotron Diffraction Analyses

X-ray powder diffraction patterns were collected on a Phillips X'pert Pro MPD powder diffractometer using a plate sample holder at room temperature. High-resolution synchrotron X-ray powder diffraction (SXPD) data were collected on Beamline I11 Diamond Light Source at the room temperature. The structure was refined using the TOPAS[5] program (CCDC number 22554850. A summary of the crystallographic data and structural determination for all the compounds is provided in Supplementary Table 4.

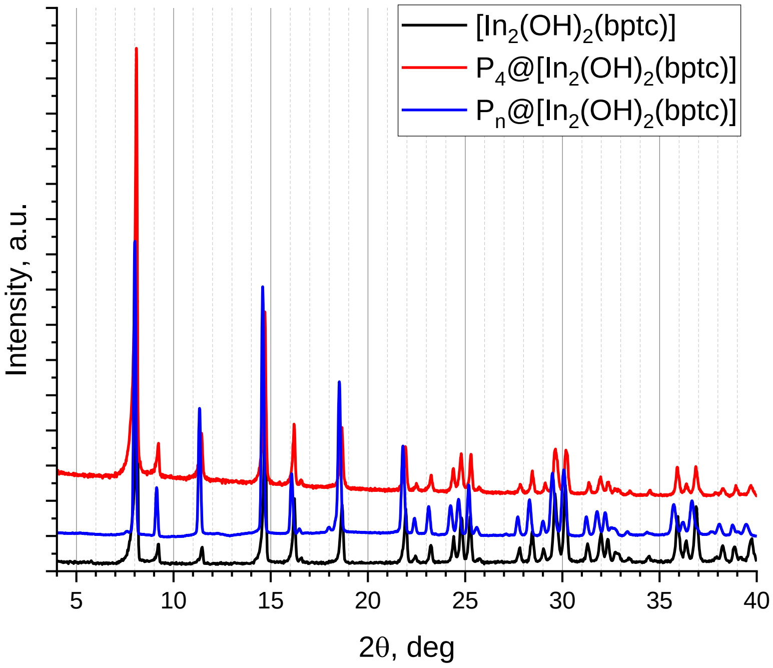

Supplementary Fig 6. PXRD patterns for MFM-300(In) (*black*), P_4@MFM-300(In) (*red*) and for the photo-polymerized adduct $(P_8)_n$@MFM-300(In) (*blue*); a.u. stands for arbitrary units.

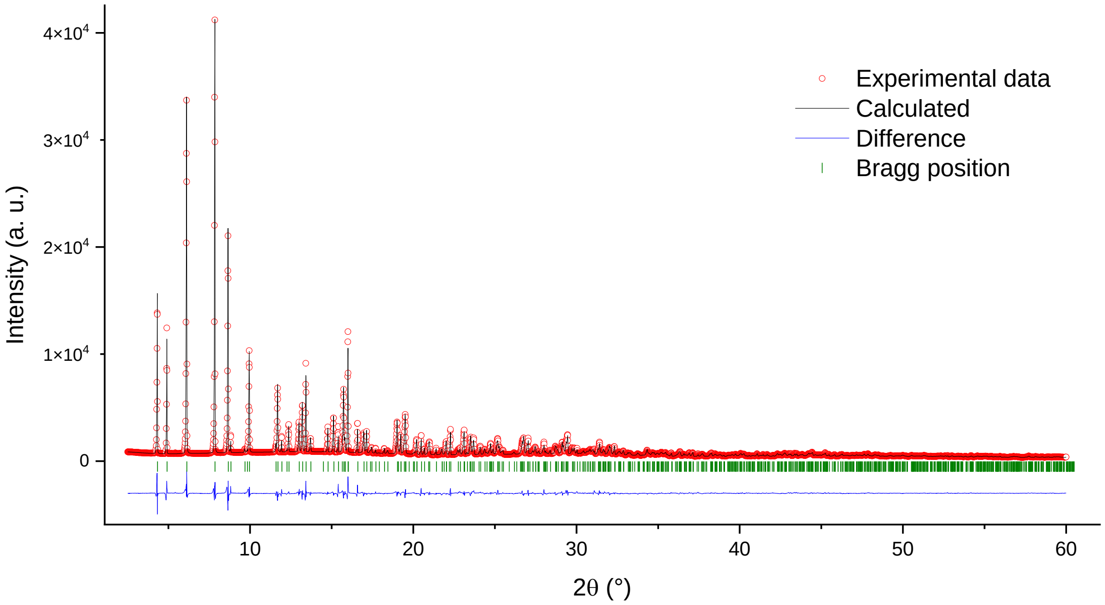

Supplementary Fig. 7. High-resolution synchrotron powder X-ray diffraction and Rietveld refinement for $(P_8)_n$@MFM-300(In); a.u. stands for arbitrary units.

Supplementary Table 4. Details of powder diffraction structure refinement of $(P_8)_n$@MFM-300(In)

	$(P_8)_n$@MFM-300(In)
Empirical Formula	$C_8H_4InO_{5.69}P_{4.89}$
T, K	298
Crystal system	Tetragonal
Space group	$I4_122$
a, Å	15.4787(2)
c, Å	12.3282(1)
V, Å^3	2953.72(9)
D_{calcd}, g/cm^3	2.057(3)
μ, mm^{-1}	3.252(4)
Wavelength, Å	0.826844
θ range, deg	1.2505 – 30.0005
Goodness of fit (GoF)	2.71736
R indices	R = 0.04540; wR = 0.06747, wR_{exp} = 0.02483.
CCDC number	2255485

Supplementary Table 5. Selected Bond Distances and Angles in $(P_8)_n$@MFM-300(In)

Bond Distances			
Bond	*d*, Å	Bond	*d*, Å
In – O(1)	2.139(8)	In – O(2)	2.05(3)
P(1) – P(3)	2.04(16)	P(1) – P(4)	2.22(17)
P(1) – P(3b)	2.29(13)	P(1) – P(4b)	1.95(16)
P(2) – P(3)	2.46(18)	P(2) – P(3b)	2.27(16)
Bond Angles			
Angle	ω, deg	Angle	ω, deg
P(3) – P(1) – P(3b)	100(5)	P(3) – P(1) – P(4)	96(5)
P(4) – P(1) – P(1b)	57(5)	P(2b) – P(1) – P(3)	62(4)

5. Details of DFT Calculations

5.1. Simulation of the Adsorption of P_4 Molecules into the Pores of MFM-300(In)

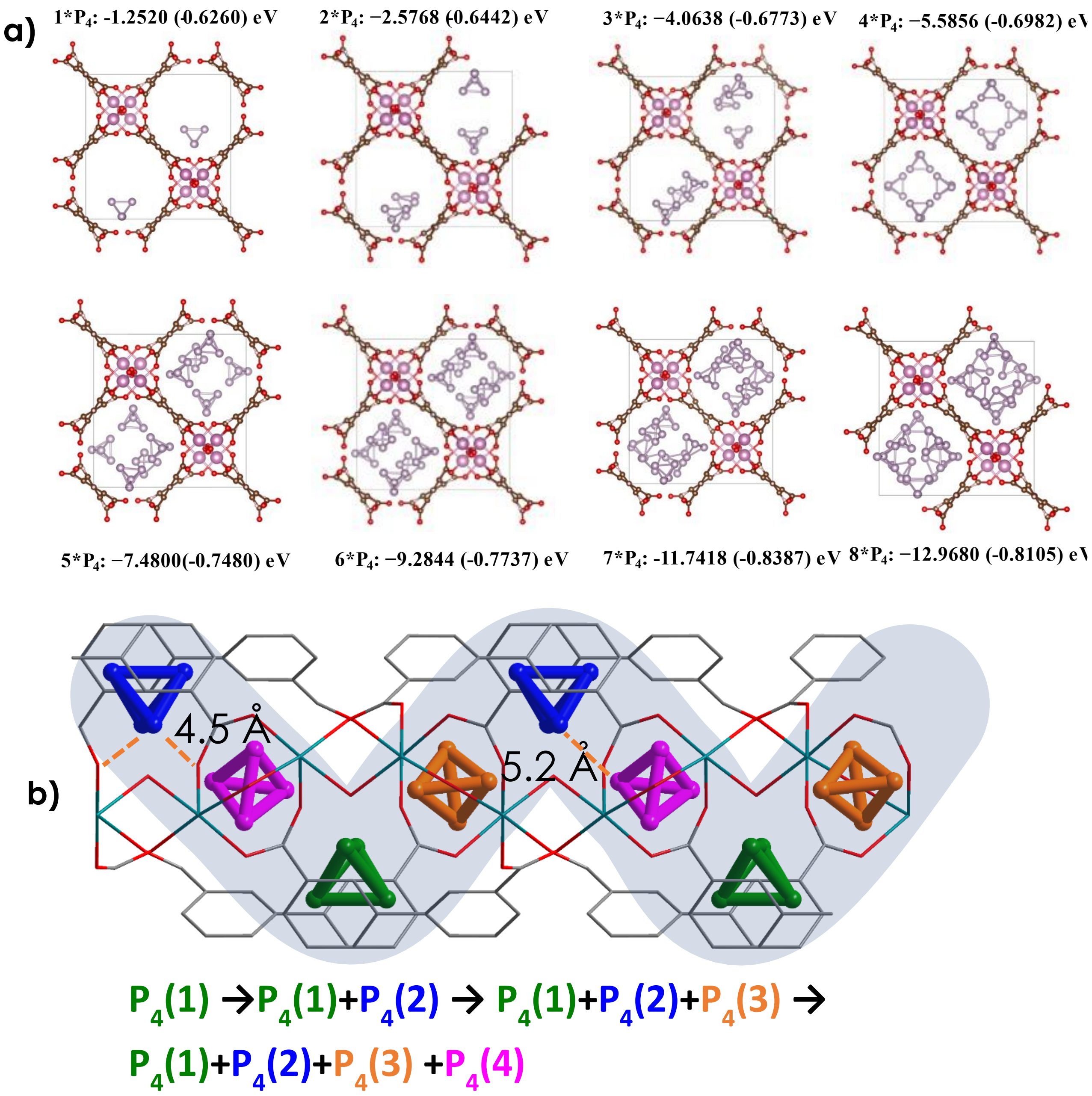

Supplementary Fig. 8. a) DFT simulation of adsorption of P_4 into MFM-300(In). The values in bold indicate the adsorption energy, with values in brackets indicating the specific total energy per phosphorus atom. b) Formation of the chain moiety of the guest P_4 molecules in P_4@MFM-300(In) simulated by *ab initio* DFT calculations. Different colours (blue, green, orange, pink) show the order of the filling of the site P_4(I) with the guest molecules.

The following diagram rationalises the contribution of guest-guest interaction to the total binding energy of the P_4 guests in the MOF. As the number of adsorbed P_4 molecules increases, the repulsion forces grow, thus decreasing the specific interaction energy in MFM-300·8P_4 (–0.8105 eV) in comparison to MFM-300·7P_4 (–0.8387 eV).

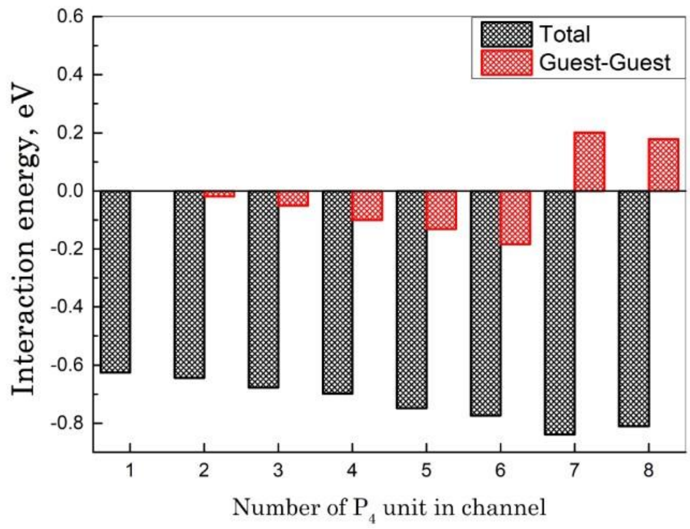

Supplementary Fig. 9. Contribution of guest-guest interaction to the total interaction energy (per P atom) at different P_4 loadings.

5.2. Geometry Optimisation of P_4@MFM-300(In) and $(P_8)_n$@MFM-300(In)

Choice of P_n chain in $(P_8)_n$@MFM-300(In) for geometry optimisation. The refined phosphorus polymer is the result of disordered simple chains (Supplementary Fig. 10). Keeping in mind that the valency of neutral P^0 should be three, there are three possible configurations of individual chains: (i) a helical chain built from a large monomer unit comprising eight "butterfly" P_4 fragments arranged with a periodicity of 3 unit cells; (ii) a helix built from "butterfly" P_4 fragments connected in a *cis-trans* fashion similar to the reported linear polyphosphorus chain within nanotubes, which is also theorised to occur in bulk red phosphorus[6]; (iii) A double helix, comprising P_8 monomers, which can be considered as a product of photo-induced cycloaddition of two butterfly fragments (Supplementary Fig. 10). Using the *ab initio* DFT calculations approach, we firstly optimised the individual chain fragments with a length of three unit cells of MFM-300(In). We then performed a full geometry optimisation for the fragment of MFM-300(In) (3 unit cells running along *c* axis) filled with the proposed chains comprising 32 phosphorus atoms with unique coordinates. The calculations show the single-helices (i) and (ii) are thermodynamically unfavourable and immediately collapse into tetrahedral P_4 molecules occupying the P_4(I) site in the pore, *i.e.*, this generates the starting material. Given the fact that in the real structure, this site has the highest occupancy this would mean, that the single chains cannot simply form spontaneously in our experiments, as the system cannot reach a minimum of total energy U_{min}, and thus the change in the free Helmholtz energy is positive $\Delta F > 0$. Only the double helix (iii) was found to be stable, the total energy of the optimised form being calculated as –16.62718 eV (–0.51960 eV per phosphorus atom). This optimised fragment is almost identical to the chain obtained from the structural data (Fig. 3c), but it is characterised by a more uniform distribution of the P–P bond length (2.237 – 2.246 Å) and more acute P…P…P folding angles (73.38°). Interestingly, the theoretically optimised double chain can be used as a blueprint for the Rietveld refinement of the powder diffraction data. In fact, a double helix with very similar geometry can be obtained by simply splitting the phosphorus atoms of the original model into two positions (Fig. 3d). Thus, the entire tubular structure observed in both single crystal and powder diffraction experiments can be described as a combination of three disordered $(P_8)_\infty$ chains, which are mutually shifted by *c*/3 distance,

where *c* is the corresponding parameter of the MFM-300(In) unit cell. The monomer is, therefore, not a single butterfly P_4 fragment, but a cycloaddition adduct P_8, which comprises two slightly distorted butterfly subunits P_4.

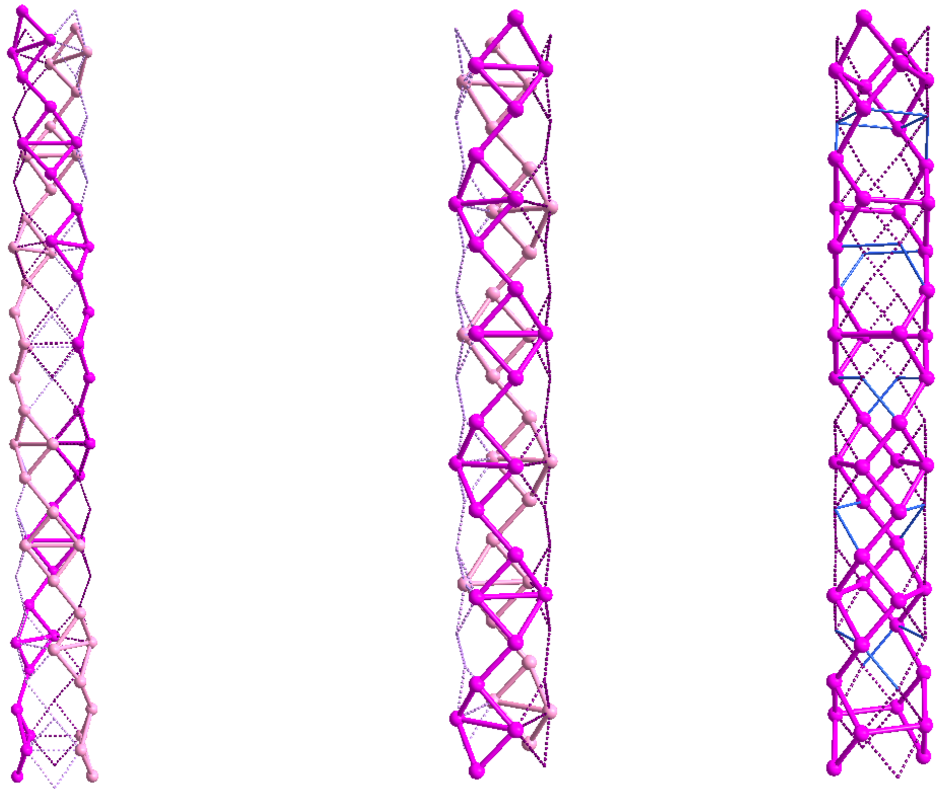

chain 1 chain 2 chain 3

Supplementary Fig. 10. Various methods of cutting the tubular structure of guest phosphorus chain in $(P_8)_n$@MFM-300(In) refined by crystallographic methods. Chains 1–3 differ in connectivity, but are built from the P atoms with the same coordinates. Different colours represent independent chains.

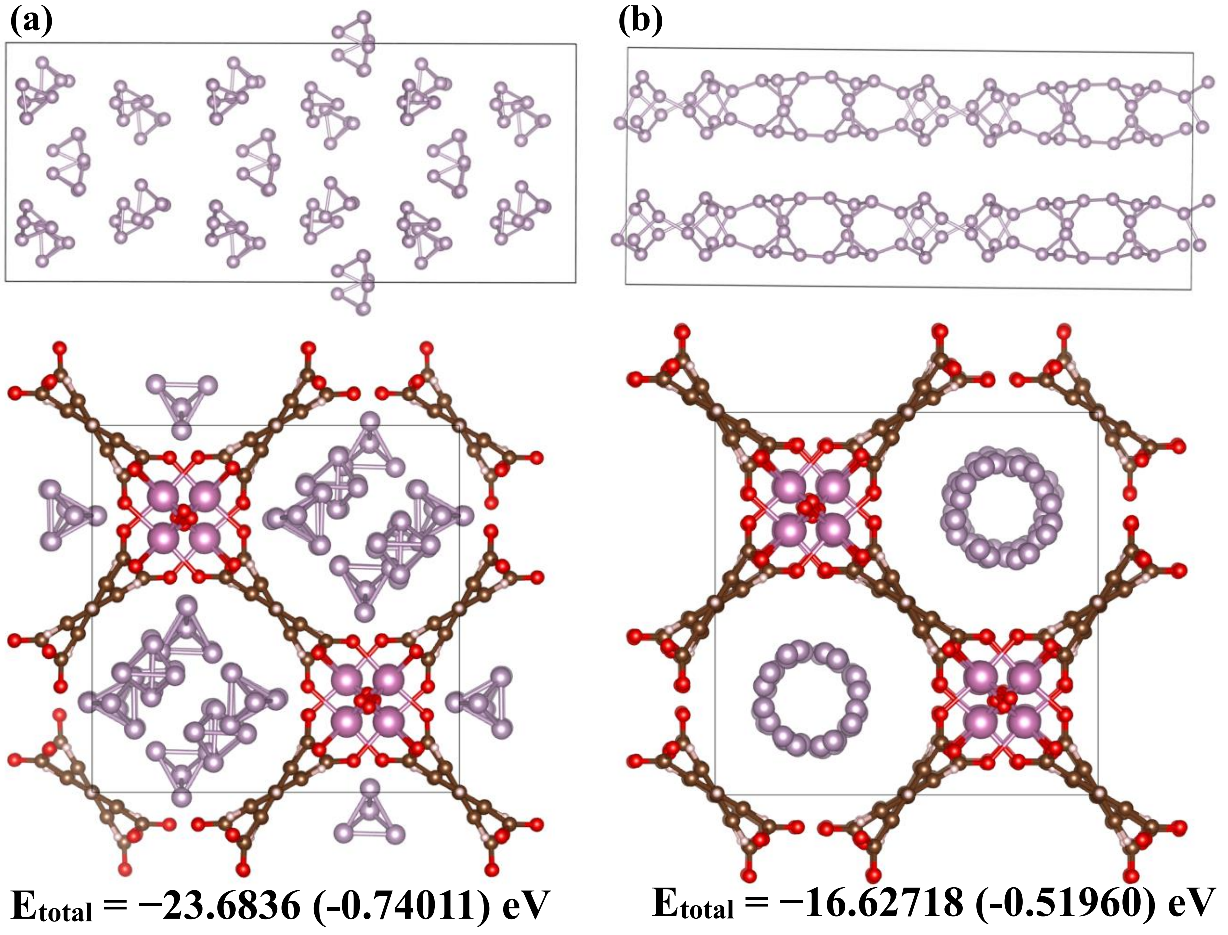

Supplementary Fig. 11. The geometrically optimised fragments of (*a*) P_4@MFM-300(In) and (*b*) $(P_8)_n$@MFM-300(In) comprising the volume of 3 unit cells. The values in brackets indicate the specific total energy of the system normalised per number of phosphorus atoms.

5.3. Calculations of Partial Charges

Five valence electrons of phosphorus atoms ($3s^2 3p^3$) were considered in the calculations. The results of the calculations for different species are stored as Source data in the file Calculations of partial charges.xls.

5.4. PDOS Calculations

To gain further understanding of electronic structure of the obtained compounds, we calculated the partial density of states (PDOS) for the optimised models of MFM-300(In), P_4@MFM-300(In) (16 P_4 molecules) and P_n@MFM-300(In) (two chains in two channels, 64 phosphorus atoms each).

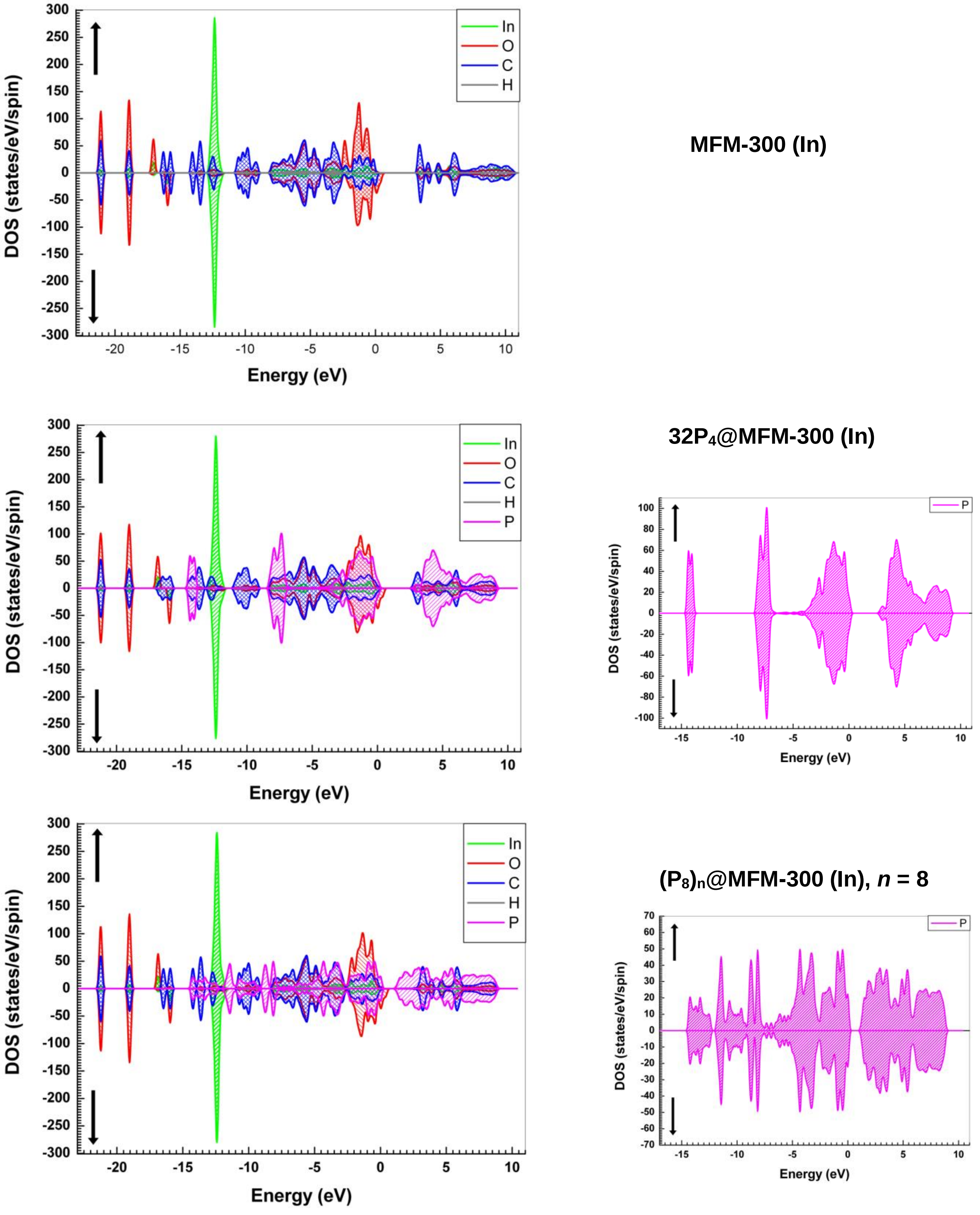

Supplementary Fig. 12. Partial density of states (PDOS) for the spin-up (↑) and spin-down (↓) of guest-free MFM-300(In), P_4@MFM-300(In) and $(P_8)_n$@MFM-300(In). The phosphorus subsystems in the corresponding inclusion compounds are shown separately.

6. Scanning Electron Microscopy

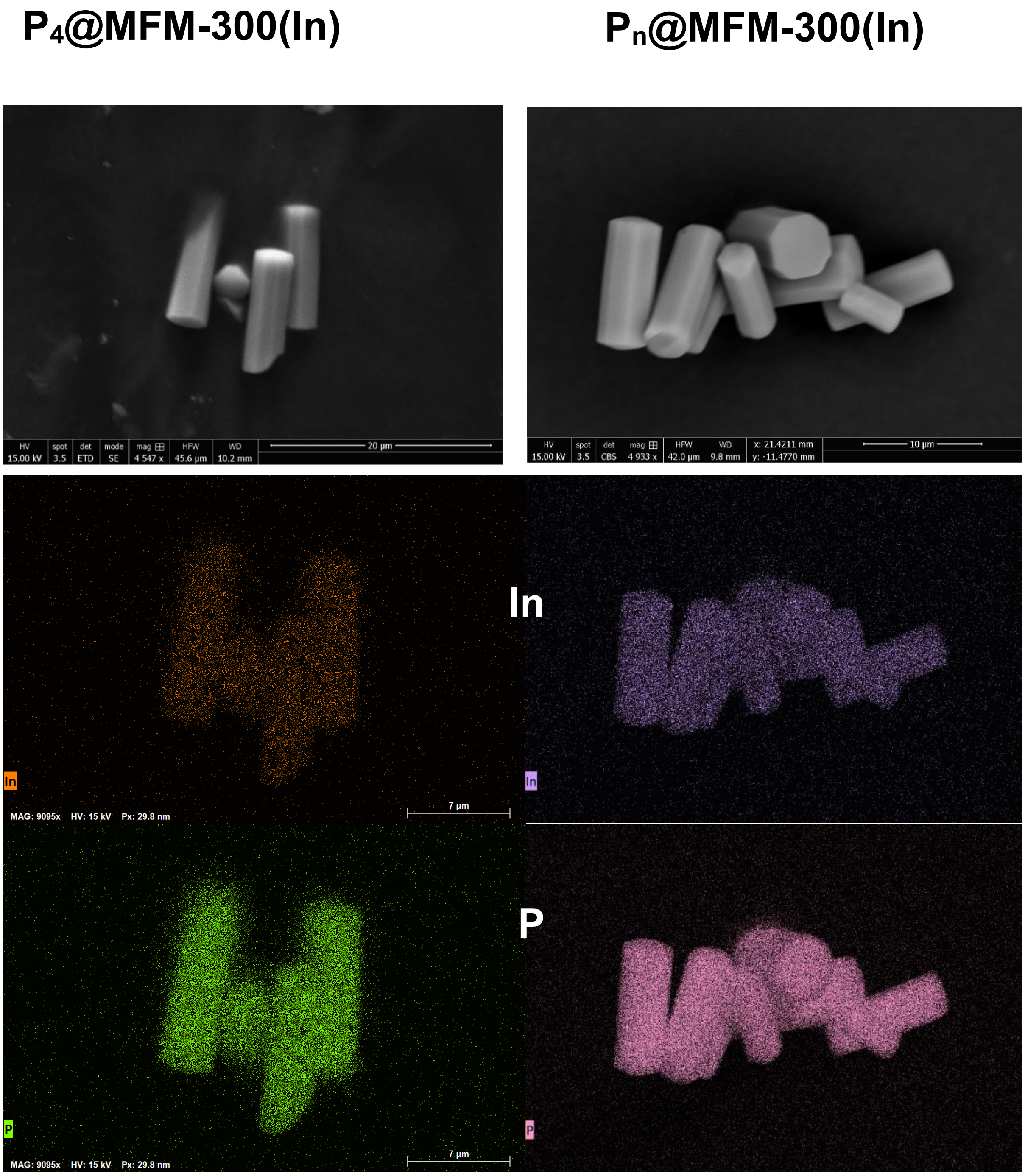

Supplementary Fig. 13. SEM images of white phosphorus and polymeric phosphorus adducts (top) and EDX mapping images for indium and phosphorus (bottom).

7. Raman Spectroscopy

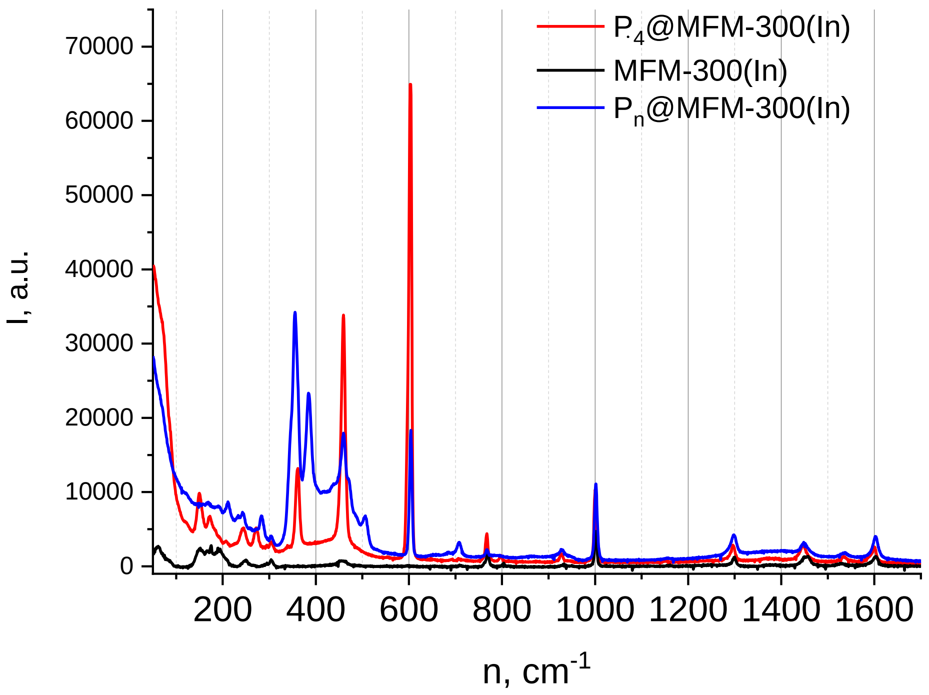

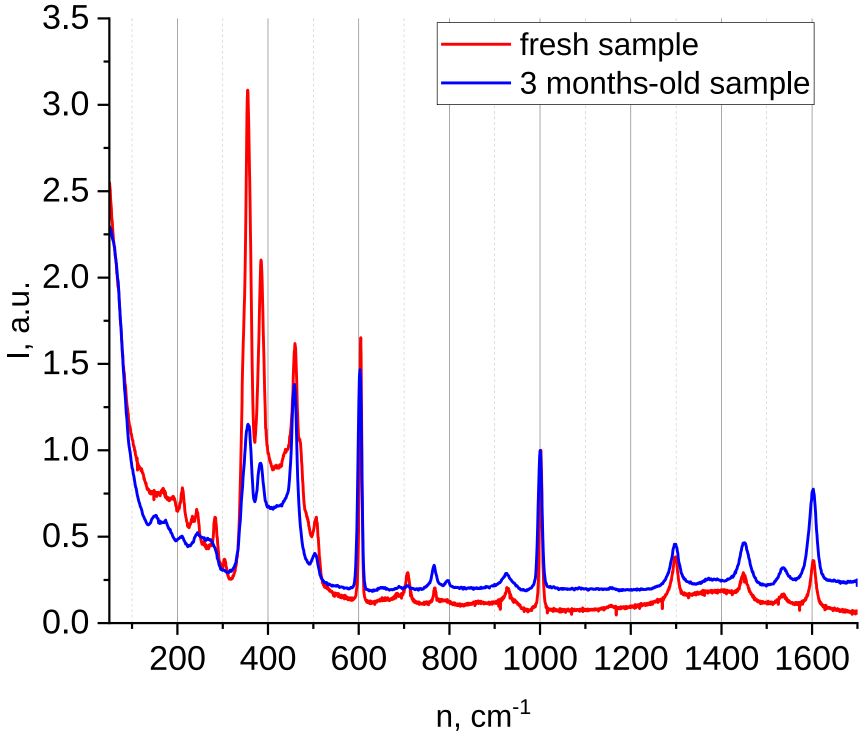

Supplementary Fig. 14. a) Raman spectra of MFM-300(In) (*black*), P_4@ MFM-300(In) (*red*) and $(P_8)_n$@MFM-300(In) (*blue*). b) Raman spectra of $(P_8)_n$@MFM-300(In) sample before (*red*) and after (*blue*) exposure to air for 3 months; a.u. stands for arbitrary units.

8. Solid-state NMR spectroscopy

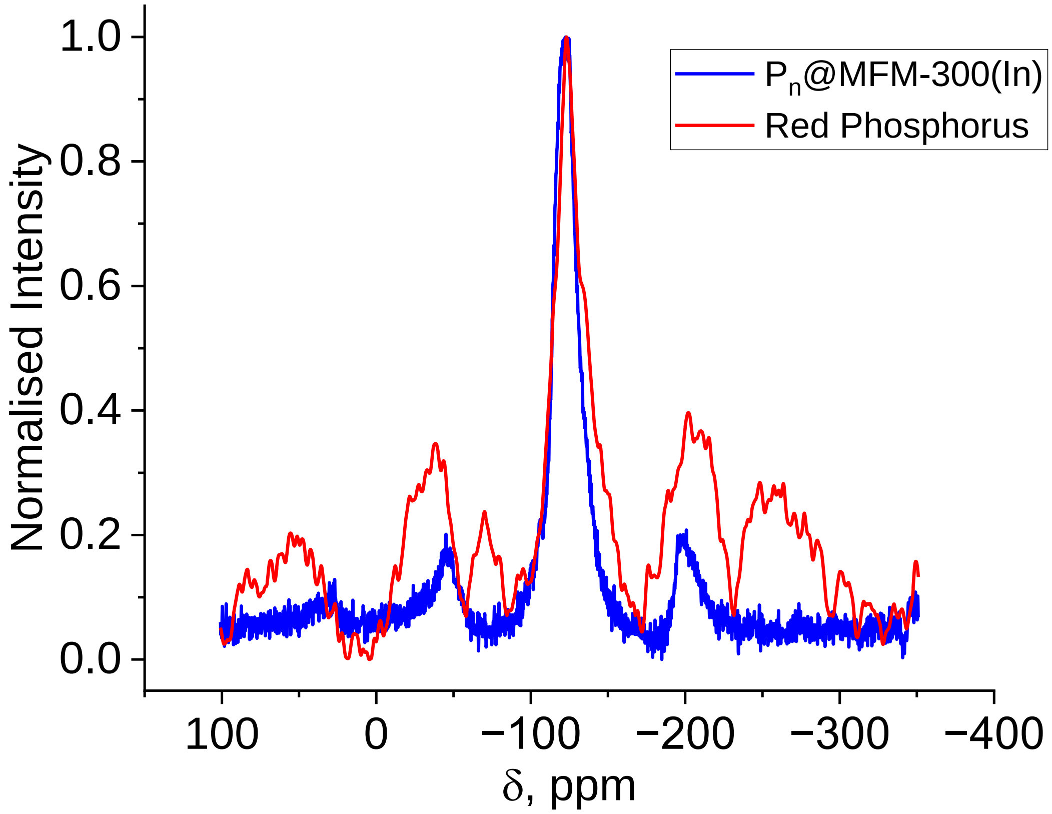

Supplementary Fig. 15. Normalised solid-state ^{31}P NMR spectra of red phosphorus (red) and $(P_8)_n$@MFM-300(In) (blue)

9. UV-Vis Spectroscopy and Calculation of Band Gap Energy.

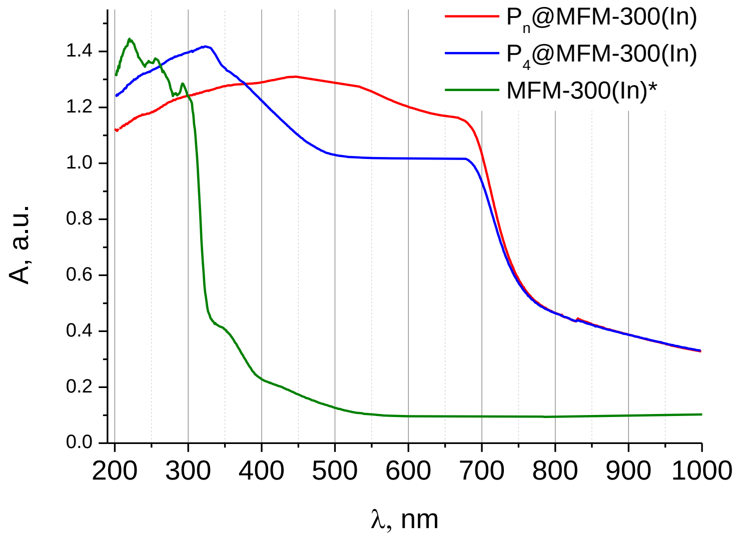

Supplementary Fig. 16. UV-Vis spectra of MFM-300(In), P_4@MFM-300(In) and $(P_8)_n$@MFM-300(In); a.u. stands for arbitrary units.

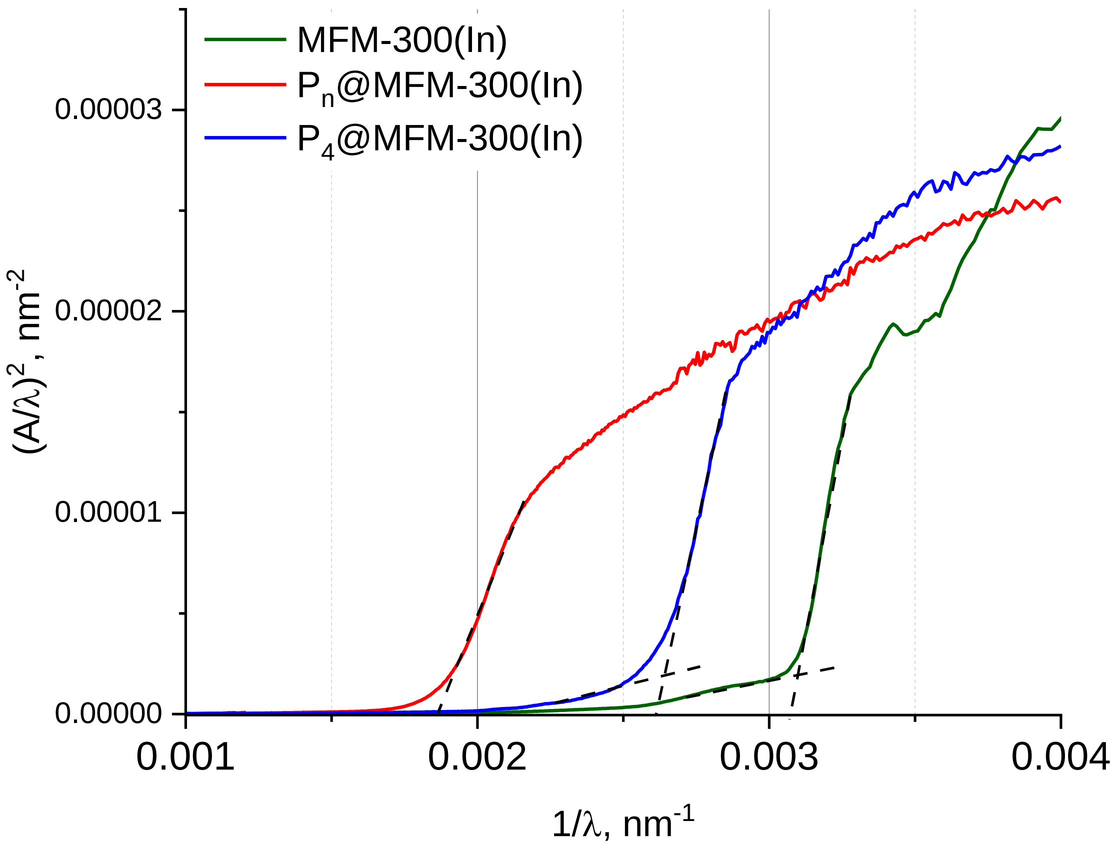

Supplementary Fig. 17. Linearisation of the UV plots for MFM-300(In), P_4@MFM-300(In) and $(P_8)_n$@MFM-300(In).

Band Gap Energy Calculations. Light adsorption by a semiconductor (*A*) can be expressed as following:

$$A = k_1\lambda\left(\frac{1}{\lambda} - \frac{1}{\lambda_g}\right)^m + k_2$$

where $k_{1,2}$ are constants accounting for the reflectance, λ is wavelength (nm), λ_g is wavelength corresponding to the band gap, and parameter $m = ½$ for symmetrical zones). By linearization of the UV spectra in $\left({}^{A}/_{\lambda}\right)^2 - {}^{1}/_{\lambda}$ coordinates (Tauc plot) the value of 1/ λ_g can be determined as a point of interception of the line with 1/ λ axis. The band gap energy E_g can be then calculated using:

$$E_g = \frac{1239.83}{\lambda_g}\,(\mathrm{eV})$$

Supplementary Table 6. Theoretical and experimental values of the band gap energy E_g

Compound	E_g, calculated / eV	E_g, found / eV
MFM-300(In)	3.0	3.9
P_4@MFM-300(In)	2.0	3.2
$(P_8)_n$@MFM-300(In)	1.0	2.3

Supplementary Table 7. Summary of selected MOFs and MOF-based inclusion compounds with experimentally determined band gap values

Compound	Band Gap, eV	Reference
ZIF-8	5.2	[7]
MOF-808	4.0	[8]
**MFM-300(In)**	**3.9**	**This work**
HKUST-1	3.5	[9]
MoS_2@ZIF-8	3.4	[7]
MOF-5	3.4	[10]
**P_4@MFM-300(In)**	**3.2**	**This work**
MIL-101(Cr)	3.0	[11]
MOF-74(Zn)	2.8	[12]
MIL-101(Fe)	2.8	[13]
[$Cu_3(HHTP)_2$]	2.7	[14]
MIL-125-NH_2	2.7	[15]
CdS@MOF-808	2.6	[8]
MIL-125-NH2@CoFe PBA	2.6	[15]
TCNQ@HKUST-1	2.4	[9]
**$(P_8)_n$@MFM-300(In)**	**2.3**	**This work**
[Fe_2(DSBDC)]	1.9	[16]
PCN-222	1.8	[17]
DMF@Mg–NDI	1.6	[18]
$CsPbI_3$@PCN-222	1.6	[17]
Cu-CAT-1 (thin films)	0.5	[19]

10. Nitrogen Adsorption Studies

To determine the Brunauer–Emmett–Teller (BET) surface area of samples, high-purity N_2 (99.999%, BOC) sorption measurements were carried out using Intelligent Gravimetric Analyser (IGA) (Hiden Isochema, Warrington, UK). Prior to the measurement, the acetone exchanged samples were degassed under vacuum (1×10^{-4} mbar) at 120 °C for 12 hours. The BET surface area of MFM-300(In) was measured as 1009 $m^2\ g^{-1}$, consistent with published data[20]. The phosphorous loaded samples were found to be non-porous to N_2 at 77 K.

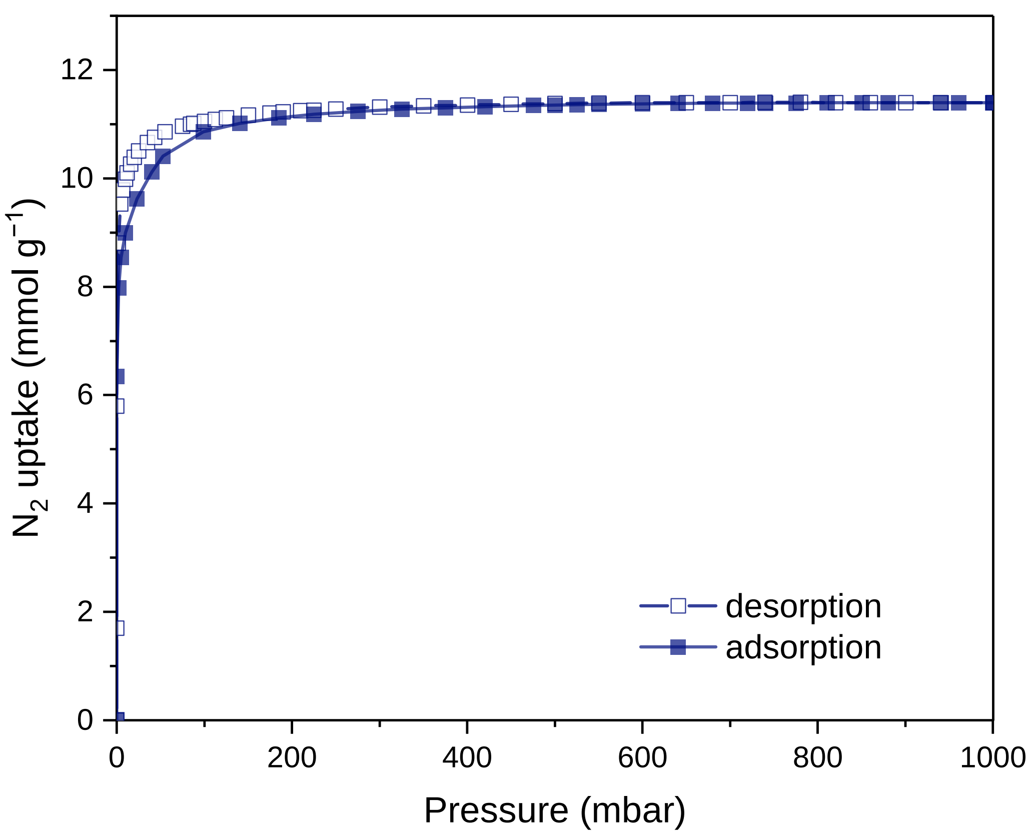

Supplementary Fig. 18. Nitrogen adsorption and desorption isotherms recorded at 77 K for MFM-300(In).

11. Electrochemical studies

11.1. Dielectric Constant and SO_2 Sensing

Dielectric constant and resistance are important characteristics of a semiconductors. Recently, red phosphorus was demonstrated to detect NO_2 by tracking the changes in the resistance/dielectric constant of a material upon exposure to the gas[21]. $(P_8)_n$@MFM-300(In) can be employed for the sensing of SO_2, which is an important air contaminant and pollutant. As we were unable to grow a thin film of this adduct, the experiment was performed on the bulk pressurised pellets, coated with silver paste.

Dielectric constant measurements were performed on the Solartron SI1260 Impedance analyser over a frequency range of 1 kHz to 1 MHz at the amplitude of 100 mV under the DC rest voltage of 0 mV at ambient conditions. The impedance analyser was connected to an electrochemical gas cell equipped with platinum current collectors. All samples were ground into fine powders and pressed into the pellets ($d \approx 0.1$ cm, $\phi = 0.8$ cm) at a pressure of 3 tons for 3 min. Ag paste was subsequently coated on the top and bottom faces of each pellet, which was transferred into the gas cell, activated under vacuum (1.0×10^{-2} mbar) at 120 – 170 °C for 12 hours before dosing with different gases. The dielectric constant was measured through the static gas dosing experiments at 0.5 bar at room temperature. Firstly, the gas cell was evacuated, then loaded with an investigated

gas by using a dosing rig. The dielectric constant was measured when the SO_2 adsorption has reached saturation, giving a constant value within the prolonged time.

The dielectric constant (ε') was calculated from the electrical capacitance (C) according to equation (2).

$$C = \varepsilon' \cdot \varepsilon_0 \cdot \frac{A}{d} \qquad (2)$$

where C is capacitance, ε' is the relative dielectric constant (the real dielectric constant of the sample), ε_0 is dielectric constant of vacuum (ε_0= 8.85×10^{-12} F/m), A is the cross-sectional area of the sample pellet, and d is the thickness of the pellet.

The experiments performed in vacuum revealed the dielectric constant of the adduct to be 7.37±0.02 (at 10 kHz), 31% higher than that of the guest-free framework (5.08±0.08 at 10kHz). Both materials were found to be stable under SO_2 (Supplementary Fig. 19) and show reversible changes of dielectric constant upon degassing/refilling the cell with SO_2 (Supplementary Fig. 20).

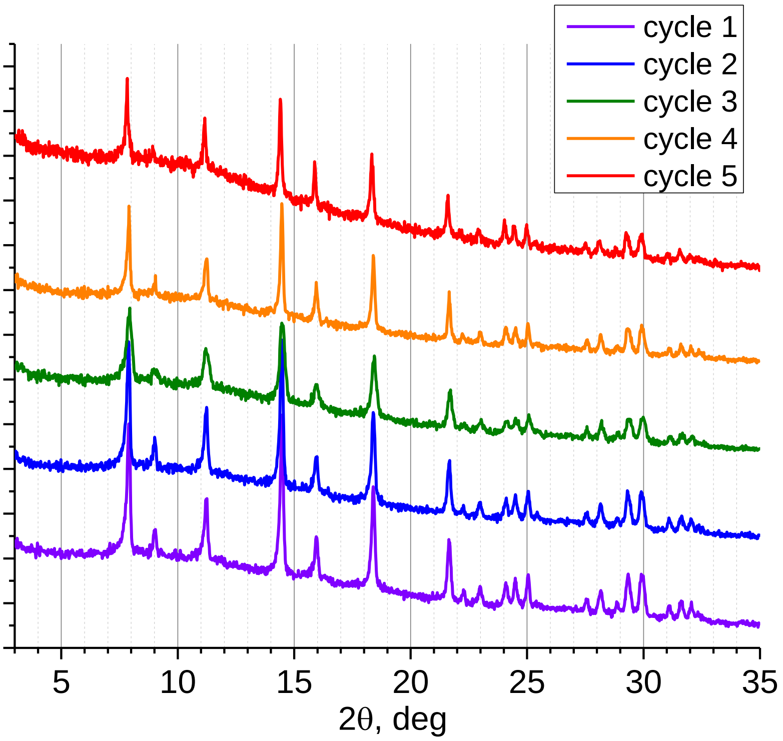

Supplementary Fig. 19. PXRD patterns of $(P_8)_n$@MFM-300(In) measured in 5 consequent SO_2 dosing/degassing cycles.

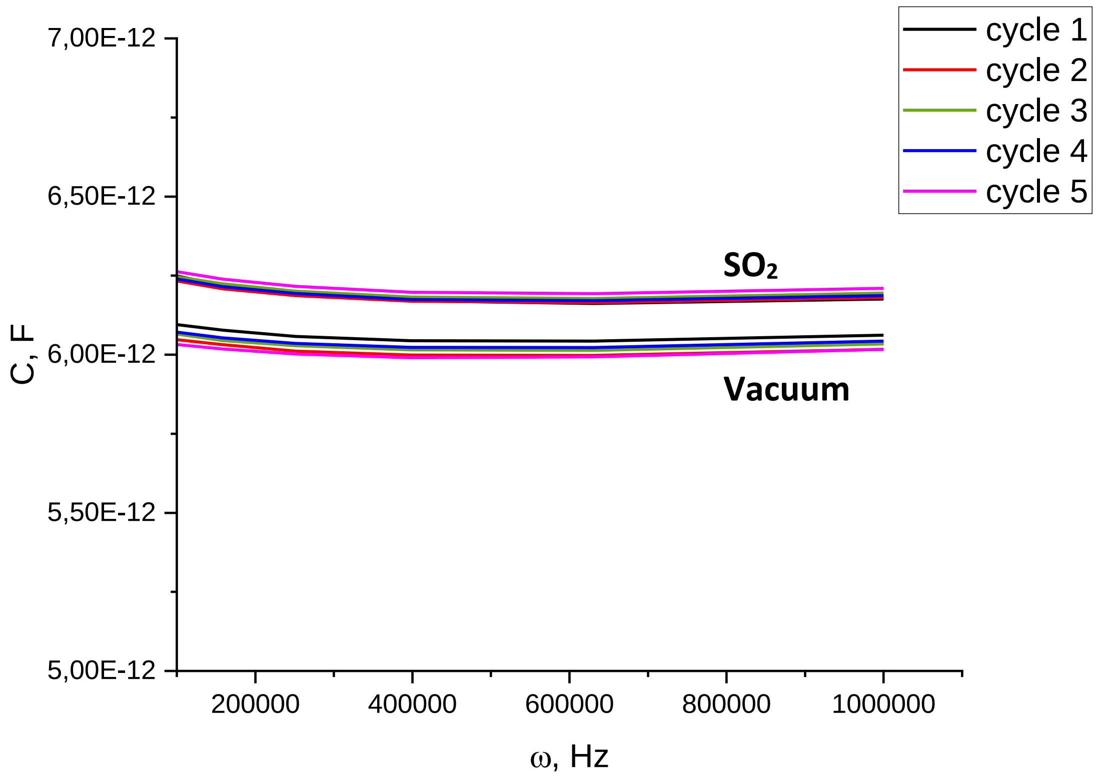

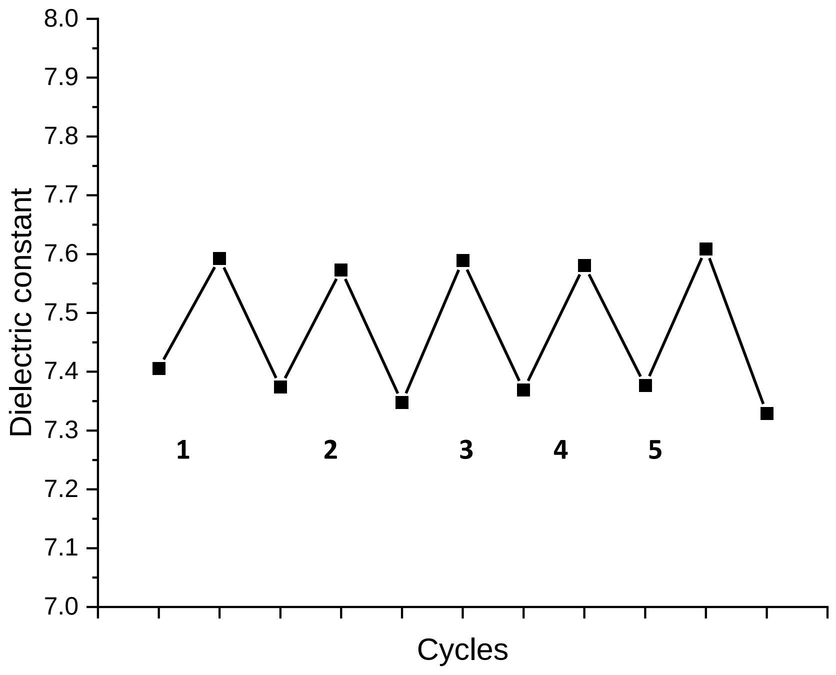

Supplementary Fig. 20. Plots of capacitance vs frequency plots measured for 5 cycles of SO_2 dosing/degassing (top); changes in the dielectric constant over 5 cycles of SO_2 dosing/degassing (bottom).

11.2. Mott-Schottky Plots

The plots show linear behaviour consistent with Mott-Schottky equation, however the plot for the phosphorus adduct features an intermediate region which can be approximated as a line with negative intercept, suggesting the presence of the surface state as opposed to the bare MOF. Studying nanowires of zinc oxide, which demonstrates very similar behaviour, Parthasarathy et al.[22] attributed these effects to surface-state-mediated mechanism of charge transfer at the electrode/electrolyte interface, while the negative slope of the plot in the charge-transfer region was caused by a distribution of surface states along the anisotropic ZnO multipods. This explanation works well for the case of $(P_8)_n$@MFM-300(In), due to the anisotropic distribution of semiconductive P_n chains which grow in one direction along the axis of the MOF channels. The dopant density N_D in $(P_8)_n$@MFM-300(In) is found to be $3.8\pm0.3\cdot10^{20}$ cm^{-3}, which is comparable to other semiconductive nanowires, for example ZnO (10^{17}–10^{18} cm^{-3})[22]. The flat band potential for $(P_8)_n$@MFM-300(In) was determined to be –0.35±0.01 V, and is below standard potential[23] $E^o(O_2(aq),e/O_2^{\bullet-})$ = –0.18±0.02 V enabling photo-catalytic oxidation in aqueous media.

a)

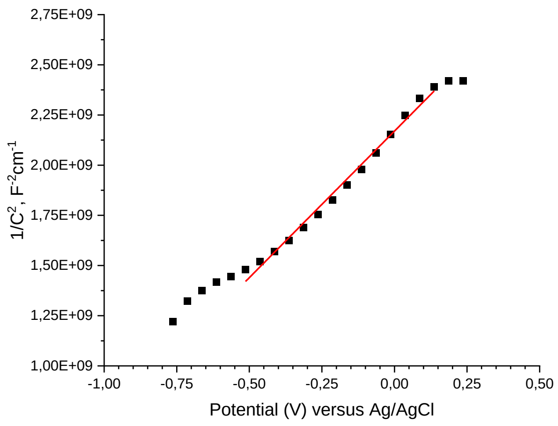

b)

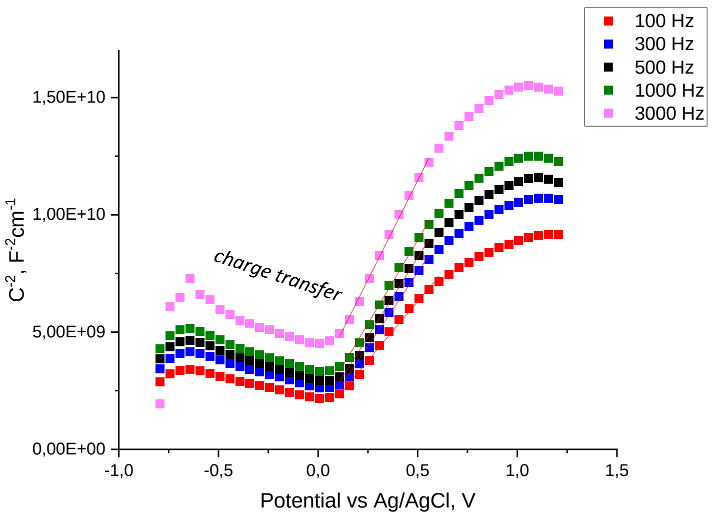

Supplementary Fig. 21. Mott-Schottky plots of a) MFM-300(In) recorded at 1kHz; b) $(P_8)_n$@MFM-300(In) measured at 100–3000 Hz *vs* Ag/AgCl electrode..

11.3 Photo-current Measurements

In comparison to bulk red phosphorus and MFM-300(In), the phosphorus adduct $(P_8)_n$@MFM-300(In) is more efficient at generating photo-current upon irradiation with visible light (Supplementary Fig. 22). The strength of the photo-current response correlates with the bandgap of the materials and decreases in the order $(P_8)_n$@MFM-300(In) > red phosphorus > MFM-300(In). The high photo-response of $(P_8)_n$@MFM-300(In) confirms its ability to separate photogenic charge carriers, which plays a crucial role in the photo-catalysis.

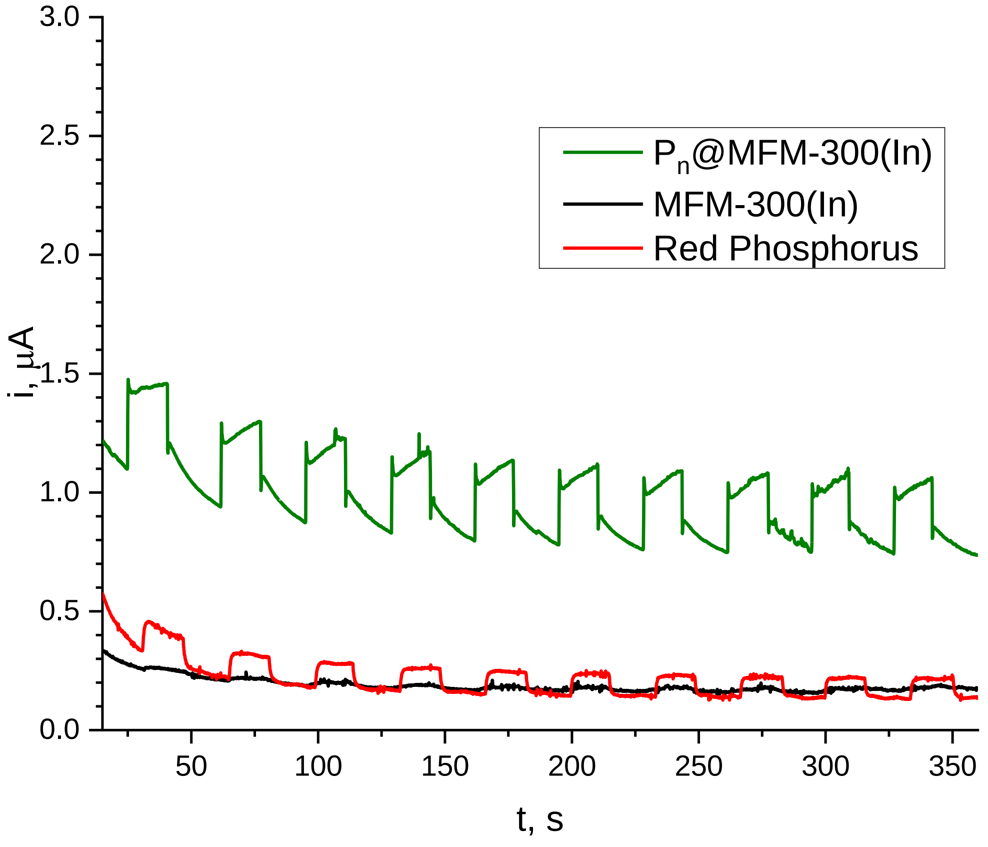

Supplementary Fig. 22. Plot of current (*i*) vs time (*t*) demonstrating the photo-current response of the MOF materials and red phosphorus.

12. Photo-luminescence Studies

Despite the strong UV and visible light absorption and its intense orange colour, the phosphorus adduct $(P_8)_n$@MFM-300(In) shows the same luminescence activity as bare MFM-300(In). Solid-state luminescent spectra of both compounds recorded upon excitation at 320 nm show a similar single broad peak with a maximum centered at 341 nm for MFM-300(In) and 346 nm for $(P_8)_n$@MFM-300(In) (Supplementary Fig. 23). The luminescence decay can be approximated to a double exponential function, giving the life-time values summarized in Supplementary Table 8. Despite different adsorption properties, both compounds have very similar luminescence life times. Since the luminescence of a solid is produced by the recombination of the photo-generated electrons and holes, the similarity in the life times of these materials suggests effective charge

carrier separation and migration. Given almost the 2-times smaller band gap in $(P_8)_n$@MFM-300(In) compared to the bare MOF, one can expect enhanced photo-catalytic performance of the former.

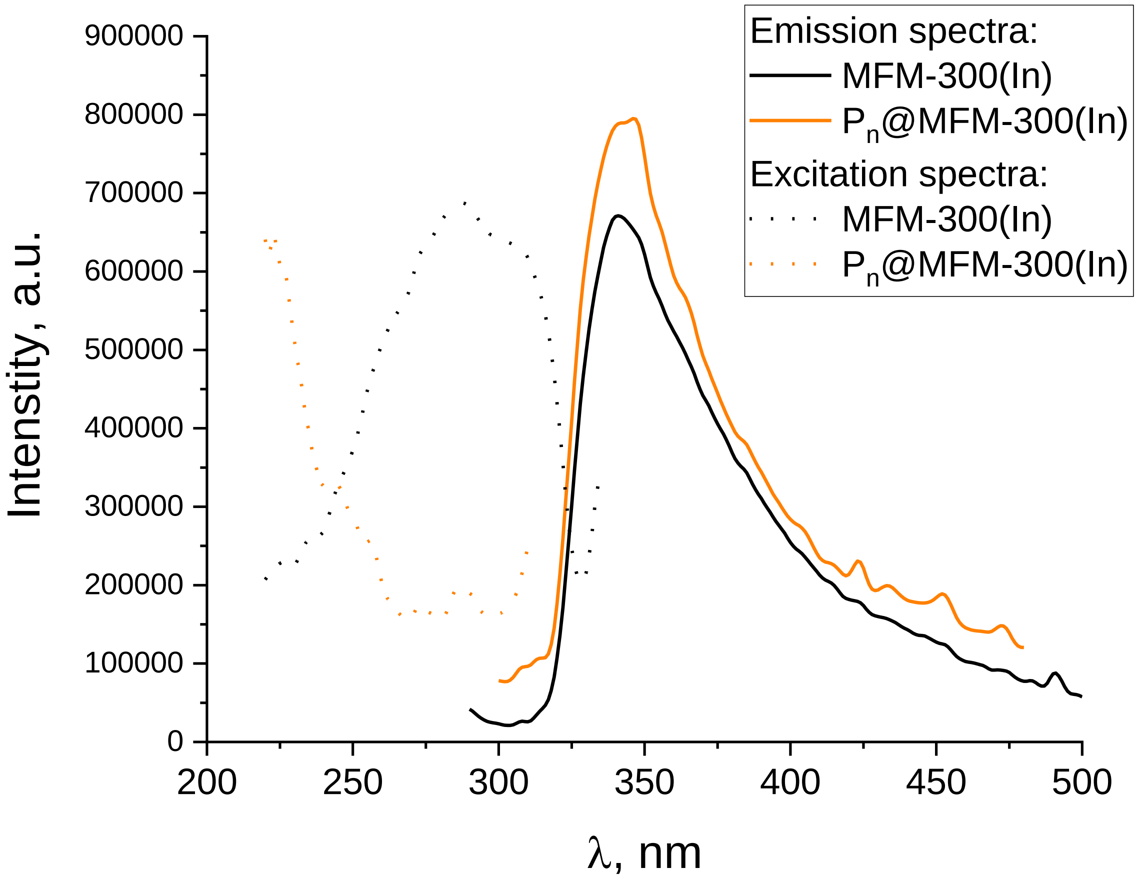

Supplementary Fig. 23. Solid-state excitation and emission spectra of guest-free framework MFM-300(In) (black) and $(P_8)_n$@MFM-300(In) (red) at λ_{ex} = 320 nm; a.u. stands for arbitrary units.

Supplementary Table 8. Luminescence life times of MFM-300 (In) and $(P_8)_n$@MFM-300(In) (λ_{ex} = 320 nm).

Sample	τ_1, ns	τ_2, ns
MFM-300(In)	0.41 ± 0.01	2.20 ± 0.01
$(P_8)_n$@MFM-300(In)	0.352 ± 0.006	3.25 ± 0.06

13. Photo-catalytic Experiments

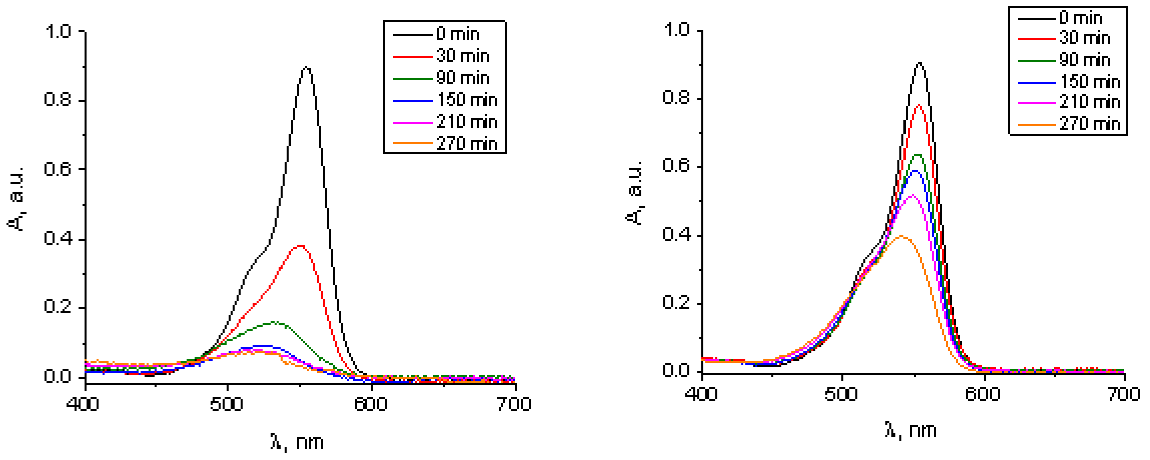

Supplementary Fig. 24. UV-Vis adsorption spectra of the reaction mixture showing the photo-degradation of Rhodamine B solution with $(P_8)_n$@MFM-300(In) (left) and MFM-300(In) (*right*); a.u. stands for arbitrary units.

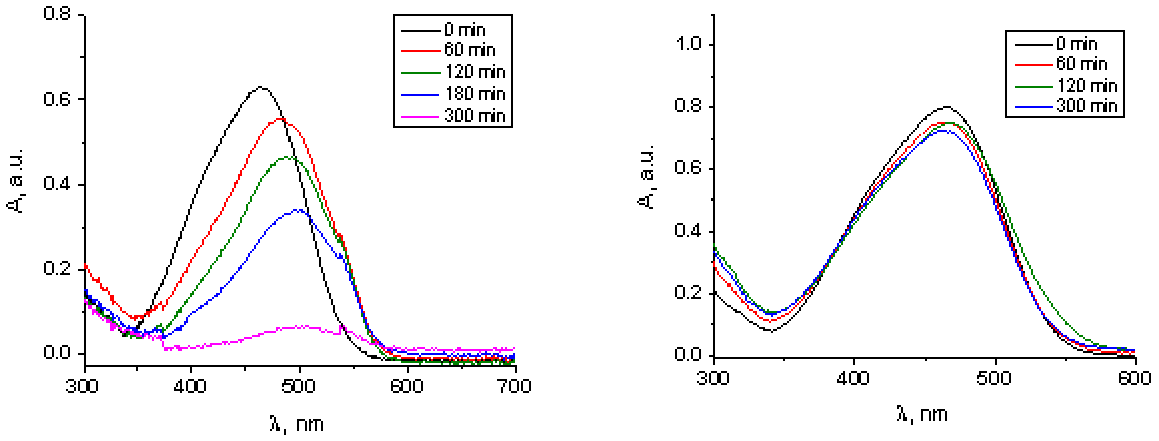

Supplementary Fig. 25. UV-Vis adsorption spectra of the reaction mixture showing the photo-degradation of Methyl Orange solution with P_n@MFM-300(In) (left) and MFM-300(In) (right); a.u. stands for arbitrary units.

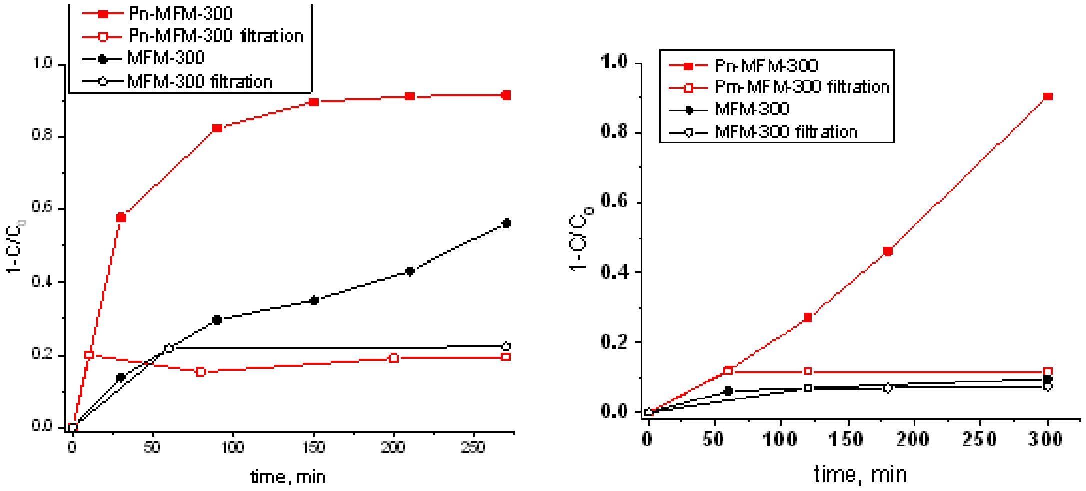

Supplementary Fig. 26. Kinetic curves: (a) Photo-oxidation of Rhodamine B with $(P_8)_n$@MFM-300(In) (red) and MFM-300(In) (black). (b) Photo-oxidation of Methyl Orange with$(P_8)_n$@MFM-300(In)) (red) and MFM-300(In) (black). The points in the kinetic curves for the filtration tests are shown as empty squares and circles, respectively.

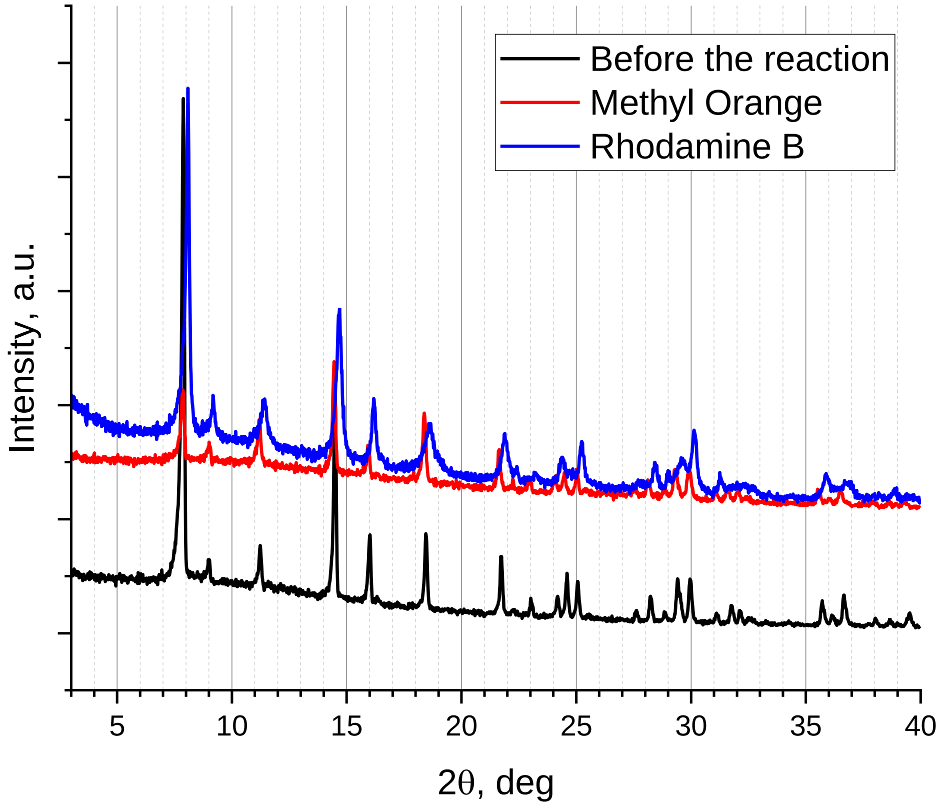

Supplementary Fig. 27. PXRD patterns for $(P_8)_n$@MFM-300(In) measured before photo-catalytic experiments (black) and after 3 cycles of reaction with Rhodamine B (blue) and Methyl Orange (red); a.u. stands for arbitrary units.

14. Supplementary References

1. CrysAlis, C.C.D. CrysAlis Red, Xcalibur PX Software, Oxford Diffraction Ltd., Abingdon, England, (2008).

2. Dolomanov, O. V., Bourhis, L. J., Gildea, R. J., Howard, J. A. K. & Puschmann, H. OLEX2 : A Complete Structure Solution, Refinement and Analysis Program. *J. Appl. Crystallogr* **42(2)**, 339–341 (2009).

3. Häser, M. Structural Rules of Phosphorus. *J. Am. Chem. Soc.* **116**, 6925–6926 (1994).

4. Hart, M., White, E.R., Chen, J., McGilvery, C.M., Pickard, C.J., Michaelides, A., Sella, A., Shaffer, M.S.P. & Salzmann, C.H. Encapsulation and Polymerization of White Phosphorus Inside Single-Wall Carbon Nanotubes. *Angew. Chem. Int. Ed.* **56**, 8144–8148 (2017).

5. TOPAS V4: General Profile and Structure Analysis Software for Powder Diffraction Data. - User's Manual. *Bruker AXS* (2008).

6. Hart, M., White, E.R., Chen, J., McGilvery, C.M., Pickard, C.J., Michaelides, A., Sella, A., Shaffer, M.S.P. & Salzmann, C.H. Encapsulation and Polymerization of White Phosphorus Inside Single-Wall Carbon Nanotubes. *Angew. Chem. Int. Ed.* **56**, 8144–8148 (2017).

7. Ren, R., Zhao, H., Sui, X., Guo, X., Huang, X., Wang, Y., Dong, Q. & Chen, J. Exfoliated Molybdenum Disulfide Encapsulated in a Metal Organic Framework for Enhanced Photocatalytic Hydrogen Evolution. *Catalysts* **9**, 89 (2019).

8. Ghosh, A., Karmakar, S., Rahimi, F. A., Roy, R. S., Nath, S., Gautam, U. K. & Maji, T. K. Confinement Matters: Stabilization of CdS Nanoparticles inside a Postmodified MOF toward Photocatalytic Hydrogen Evolution. *ACS Appl. Mater. Interfaces* **14**, 25220 – 25231 (2022).

9. Talin, A. A., Centrone, A., Ford, A. C., Foster, M. E., Stavila, V., Haney, P., Kinney, R. A., Szalai, V., El Gabaly, F., Yoon, H. P., Léonard, F. & Allendorf, M. D. Tunable Electrical Conductivity in Metal-Organic Framework Thin-Film Devices. *Science* **343**, 66 – 69 (2014).

10. Alvaro, M., Carbonell, E., Ferrer, B., Llabrés i Xamena, F. & Garcia, H. Semiconductor Behavior of a Metal-Organic Framework (MOF). *Chem. Eur. J.* **13**, 5106 – 5112 (2007).

11. Su, S., Li, X., Zhang, X., Zhu, J., Liu, G., Tan, M., Wang, Y. & Luo, M. Keggin-type SiW_{12} Encapsulated in MIL-101(Cr) as Efficient Heterogeneous Photocatalysts for Nitrogen Fixation Reaction. *J. Colloid and Interface Science* **621**, 406 – 415 (2022).

12. Botas, J. A., Calleja, G., Sanchez-Sanchez, M. & Orcajo, M. G. Effect of Zn/Co Ratio in MOF-74 Type Materials containing Exposed Metal Sites on their Hydrogen Adsorption Behaviour and on their Band Gap Energy. *Int. J. Hydrogen Energy* **26**, 10834–10844 (2011).

13. Li, Y., Wang, X., Duan, Z., Yu, D., Wang, Q., Ji, D. & Liu, W. Zn/Co-ZIFs@MIL-101(Fe) Metal–Organic Frameworks are Effective Photo-Fenton Catalysts for RhB Removal. *Sep. Purif. Technol*. **293**, 121099 (2022).

14. De Lourdes Gonzalez-Juarez, M., Flores, E., Martin-Gonzalez, M., Nandhakumar, I. & Bradshaw, D. Electrochemical Deposition and Thermoelectric Characterisation of a Semiconducting 2-D Metal-Organic Framework Thin Film. *J. Mater. Chem. A* **8**, 13197–13206 (2020).

15. Yuan, L., Zhang, C., Zou, Y., Bao, T., Wang, J., Tang, C., Du, A., Yu, C. & Liu, C. A S-Scheme MOF-on-MOF Heterostructure. *Adv. Funct. Mater.* **33**, 2214627 (2023).

16. Li, J., Kumar, A., Johnson, B.A. et al. Experimental Manifestation of Redox-Conductivity in Metal-Organic Frameworks and its Implication for Semiconductor/insulator Switching. *Nat Commun* **14**, 4388 (2023).

17. Xia, Z., Shi, B., Zhu, W., Xiao, Y. & Lü, C. Binary Hybridization Strategy toward Stable Porphyrinic Zr-MOF Encapsulated Perovskites as High-Performance Heterogeneous Photocatalysts for Red to NIR Light-Induced PET-RAFT Polymerization. *Adv. Funct. Mater.* **32**, 2207655 (2022).

18. Mallick, A., Garai, B., Addicoat, M.A., Petkov, P. St., Heine, T. & Banerjee, R. Solid State Organic Amine Detection in a Photochromic Porous Metal Organic Framework. *Chem. Sci.* **6**, 1420 – 1425 (2015).

19. Rubio-Giménez, V., Galbiati, M., Castells-Gil, J., Almora-Barrios, N., Navarro-Sánchez, J., Escorcia-Ariza, G., Mattera, M., Arnold, T., Rawle, J., Tatay, S., Coronado, E. & Martí-Gastaldo, C. Bottom-Up Fabrication of Semiconductive Metal–Organic Framework Ultrathin Films. *Adv. Mater.* **30**, 1704291 (2018).

20. Savage, M., da Silva, I., Johnson, M., Carter, J. H., Newby, R., Suyetin, M., Besley, E., Manuel, P., Rudić, S., Fitch, A. N., Murray, C., David, W. I. F., Yang, S. & Schröder, M. Observation of Binding and Rotation of Methane and Hydrogen within a Functional Metal–Organic Framework. *J. Am. Chem. Soc.* **138**, 9119–9127 (2016).

21. Zhu, Q., Wang, H., Yang, J., Xie, C., Zeng, D. & Zhao, N. Red Phosphorus: an Elementary Semiconductor for Room Temperature NO_2 Gas Sensing. *ACS Senc.* **3**, 2629–2636 (2018).

22. Parthasarathy, M., Ramgir, N.S., Sathe, B.R., Mulla, I.S. & Pillai, V.K. Surface-State-Mediated Electron Transfer at Nanostructured ZnO Multipod/Electrolyte Interfaces. *J. Phys.Chem. C* **111**, 13092–13102 (2007).

23. Armstrong, D.A., Huie, R.E., Koppenol, W.H., Lymar, S.V., Merényi, G., Neta, P., Ruscic, B., Stanbury, D.M., Steenken S. & Wardman, P. Standard Electrode Potentials Involving Radicals in Aqueous Solution: Inorganic Radicals (IUPAC Technical Report). *Pure Appl. Chem.* **87**, 1139–1150 (2015).
